# Ex ante and ex post effects of hybrid index insurance in Bangladesh^☆^

**DOI:** 10.1016/j.jdeveco.2018.09.003

**Published:** 2019-01

**Authors:** Ruth Vargas Hill, Neha Kumar, Nicholas Magnan, Simrin Makhija, Francesca de Nicola, David J. Spielman, Patrick S. Ward

**Affiliations:** a The World Bank, Washington, DC, USA; b International Food Policy Research Institute, Washington, DC, USA; c University of Georgia, Athens, GA, USA; d Duke Kunshan University, Kunshan, Jiangsu Province, China

**Keywords:** Index insurance, Risk and uncertainty, Agriculture, Bangladesh

## Abstract

This study assesses both the demand for and effectiveness of an index insurance product designed to help smallholder farmers in Bangladesh manage crop production risk during the monsoon season. Villages were randomized into either an insurance treatment or a comparison group, and discounts and rebates were randomly allocated across treatment villages to encourage insurance take-up and to allow for the estimation of the price-elasticity of insurance demand. Among those offered insurance, we find demand to be fairly price elastic, with discounts significantly more successful in stimulating demand than rebates. Purchasing insurance yields both *ex ante* risk management effects as well as *ex post* income effects on agricultural production practices. The risk management effects lead to an expansion of cultivated area with concomitant increases in agricultural input expenditures during the monsoon season. The income effects lead to more intensive rice production during the subsequent dry season, with more intensive use of both irrigation and fertilizers, resulting in higher yields and higher total rice production.

## Highlights

• We evaluate a hybrid index insurance product in northwestern Bangladesh.
• Impacts include both *ex ante* risk mitigation effects and *ex post* income effects.
• Risk mitigation effects: expanded, diverse production; higher investments in inputs.
• Income effects: expanded rice production during the dry season.
• Income effects: intensified use of fertilizers; higher yields.

## 1 Introduction

Agricultural production in developing countries is fraught with various sources of risk. The type and severity of these risks varies by crop or farming system, agroecological conditions, and the policy and institutional settings (Hazell et al., 1986). A seemingly ubiquitous source of agricultural risk is production risk due to weather uncertainty and variability, particularly those associated with deficient rainfall. There are various strategies to mitigate such drought risks, including investments in infrastructure (e.g., irrigation facilities), technological innovations (e.g., drought-tolerant cultivars), crop management practices (e.g., changes to the timing of production activities), and financial instruments (e.g., credit or insurance). Unfortunately most of these strategies are often either not available or not feasible for many resource-constrained farmers in developing countries. Consequently, droughts often result in lower crop productivity, while the *risk* of drought disincentivizes otherwise optimal investments in new technologies and modern farm inputs (Sandmo, 1971; Quiggin, 1992; Ramaswami, 1992). Though these various management decisions may reduce both the level and variability income or consumption in the short run, they do so at the expense of constrained long-run economic growth.

In this paper we focus on insurance, and assess the degree to which insurance markets can be developed for resource-constrained farmers in low-income settings and incentivize optimal agricultural investments. Conventional indemnity-based crop insurance – which insures farmers against assessed crop losses – is problematic due to asymmetric information (resulting in moral hazard and adverse selection) and high transaction costs (Hazell, 1992; Just et al., 1999; Morduch, 2006; Barnett et al., 2008; Carranco, 2017; Gunnsteinsson, 2017). Index insurance, on the other hand, provides insurance coverage on the basis of observed indices, typically derived from weather conditions measured at a local weather station or average yields recorded in a given area, rather than directly assessed individual yield or profit losses (Morduch, 2006; Giné et al., 2008; Karlan and Morduch, 2009). As index-based insurance does not require verification or assessment of losses at the farm level, it minimizes asymmetric information and drastically reduces the delays and costs associated with conventional crop insurance, including both administrative and re-insurance costs (Barnett and Mahul, 2007). For these reasons, many development practitioners and policymakers are cautiously optimistic about the potential for index insurance to stimulate agricultural investment and productivity (Alderman and Haque, 2007; Hazell et al., 2010).

Because payouts are made on the performance of an index, however, they are not always commensurate with the losses that a farmer has experienced, and this leads to basis risk – the risk that the farmer experiences a loss and receives no insurance payout because it is not a loss that is reflected by the index (Clarke, 2016). Basis risk, when combined with other factors such as liquidity constraints, limited familiarity with the insurance principles, and lack of trust in the insurance provider, can constrain demand (Cole et al., 2013a,b; Giné et al., 2008; Giné and Yang, 2009; Hill et al., 2016). As a result, many index insurance programs piloted to date have had limited success (Binswanger-Mkhize, 2012). When insurance is adopted at reasonable scale, however, much of the emerging evidence suggests that it is successful in encouraging productive investments (Karlan et al., 2014; Elabed and Carter, 2015; Mobarak and Rosenzweig, 2013; Berhane et al., 2014).

This study assesses both the demand for and effectiveness of an innovative hybrid index insurance product designed to help smallholder farmers in Bangladesh manage risk to crop yields and the increased production costs associated with drought during the monsoon season. The product we evaluate incorporates an area yield index, reflecting the common use of such indices in most index-insurance products sold in Asia to cover many different sources of risk (Clarke, 2016; Cai, 2016). However, the policy also provided payouts for prolonged dry spells. While most observers might not think of Bangladesh as being particularly prone to droughts, in fact, droughts cause significant damage to an estimated 2.32 million hectares of the transplanted rice crop (the *t. aman* crop) cultivated during the monsoon season, with serious nationwide droughts occurring roughly once every five years (Ramamasy and Baas, 2007).^1^ The widespread increase in the availability of irrigation in recent years has allowed Bangladeshi farmers to mitigate the impact of drought on production, but the use of irrigation to do so is costly, such that rainfall deficiencies can ultimately result in increases in the costs of production, in addition to any residual impacts on yields.

Among the households interviewed for the present study, for example, irrigation comprises 14 percent of out of pocket expenses on average. To address these risks, the index insurance product that we evaluate was designed to provide payouts based on the number of consecutive dry days that were observed during the monsoon season. This index is correlated with costs of production if on average households mitigate weather shocks through irrigation, or with yields if on average households are not fully able to mitigate weather shocks.

The randomized controlled trial (RCT) described here was designed to evaluate a local nongovernmental organization's (NGO) index insurance pilot program in Bogra district in northwestern Bangladesh during the 2013 monsoon season. The insurance product was intended to cover production risks on a 10 decimal (0.1 acre) plot of land during the season. Discounts and rebates were randomly allocated to villages to encourage insurance take-up, to allow the price-elasticity of demand to be calculated, and to evaluate the trade-off between providing discounts and rebates.

A priori, one might expect that discounts would be preferred to rebates given they help address liquidity constraints at the time of insurance purchase. Additionally, there is evidence from various studies in several developing countries that suggest individuals value the present more than the future, and would therefore prefer the immediate benefit of a discount to the delayed benefit of a rebate (Duflo et al., 2011). Along similar lines, individuals may prefer the discount because there is more certainty associated with a discount now, whereas the promise of a rebate in the future entails some uncertainty. Interestingly, however, despite the uncertainty, this promise of a future payment may be alluring for some farmers. In the context of insurance, rebates provide a certain payout in the future regardless of whether the insurance pays out, and this has been shown to be preferred in Burkina Faso (Serfilippi et al., 2016).

We find insurance demand to be moderately price elastic. The incentives offered were quite high, and as a result, a large proportion of households purchased at least one unit of insurance. Discounts were significantly more successful in stimulating demand than rebates, which entail a sizable lag between when the purchase is made and when the benefits of the incentive are realized. The price elasticity implied by the results suggests that there would need to be a 15 percent discount or a 33 percent rebate relative to the actuarially fair price of insurance in order to observe purchases of a single unit of insurance. It is possible that the discounts required to sustain demand would fall over time as farmers came to know and value the product (e.g., Cole et al., 2014), but this remains to be seen as we do not know enough about how demand may change over time as people learn about the product. Despite the preference for discounts in aggregate, we find some significant heterogeneity in demand responses to a rebate, suggesting that some individuals, particularly those that are especially risk-averse or sensitive to basis risk, may implicitly view the rebate as a commitment savings mechanism that can offset the costs of insurance contract nonperformance, especially if they experience an on-farm loss and yet are not indemnified by the insurance.

We also find, consistent with theory, that insurance encouraged farmers to take on greater risk, particularly through expanding area under higher-value crops and through investments in risk-increasing agricultural inputs during the monsoon season. At the same time, insurance also increased use of irrigation to mitigate the yield impact of the long dry spell that was recorded in the 2013 monsoon season. The dry spell in the 2013 monsoon season was long enough to trigger an insurance payment which disbursed prior to land preparation for the subsequent *boro* rice-growing season.^2^ No insurance was offered to farmers in the post-monsoon dry season, but the disbursement of insurance payments provided farmers with a liquidity injection that led to increased investments in risk-increasing modern agricultural inputs related to *boro* production. While there was no significant effect on *aman* rice production or productivity during the monsoon season, we find that the increased investment in modern inputs during the dry season led to a roughly 8 percent increase in *boro* rice production.

The remainder of this paper is organized as follows. Section 2 provides a brief literature review on the determinants of insurance demand and the impacts of index-based insurance – particularly on investments in modern agricultural inputs. Section 3 describes the experimental context, the insurance product, and our experimental design. Section 4 presents the empirical results on determinants of insurance demand and in Section 5 we present findings on the impact of insurance on agricultural input use. In Section 6, we offer some concluding thoughts and discuss the policy implications of our findings.

## 2 Review of the literature on the impacts of insurance and determinants of insurance demand

Insurance transfers income from high-income states of the world to low-income states of the world, increasing utility for risk-averse individuals. Yet indemnity-based crop insurance programs in many developing countries have struggled, arguably due to poor contract performance, asymmetric information, high transaction costs, and high exposure to covariate risks (Barnett et al., 2008; Hazell, 1992; Carter et al., 2016; Binswanger-Mkhize, 2012). To circumvent some of these impediments, public policymakers and development practitioners have turned to index-based insurance programs, which base payments on the performance of some transparent, easy-to-measure index relative to some benchmark.

One of the benefits of insurance is that it is expected to increase average incomes for farm households by affecting production decisions. There has long been a theoretical understanding that risks act as an impediment to what would otherwise be profit-maximizing investments. While Sandmo (1971) is primarily concerned with producer behavior under output price risk, production risk may arguably have a greater impact on production decisions in the agricultural sector, and is almost certainly the most salient source of risk faced by smallholder farmers in developing countries. Carter et al. (2016) show that index insurance can increase the adoption of high-return risk-increasing technologies when the risks are well-covered by the index.

Index-based insurance products have several advantages over traditional crop insurance (e.g., Miranda, 1991). First, payments are based on index triggers that are typically easy to observe and measure, making the index more transparent to the insured, minimizing asymmetric information between the insured and insurer, and reducing the probability of adverse selection and moral hazard (Clarke et al., 2015). This allows for payments to be calculated easily and distributed in a timely manner. Additionally, because insurance payments are based on an index rather than loss adjustments calculated for each farm that is insured, operating and administrative costs are significantly lower than those of other types of agricultural insurance (Barnett et al., 2008).

Despite these benefits, however, index-based insurance has a considerable disadvantage. Farmers only receive compensation when the level of the index relative to some threshold triggers payouts. Since most indices are tied to observable weather outcomes which are only imperfectly correlated with on-farm losses (e.g., Rosenzweig and Binswanger, 1993), there is a nontrivial probability that farmers will not be compensated even when they realize significant on-farm losses. Perils unrelated to the index such as soil conditions, pest and disease infestations, and farmer illness also affect yields. The risk that a farmer may incur a large loss and still not receive any payment from the insurance contract is referred to as basis risk, and has been shown to pose a major deterrent to index insurance uptake (Clarke, 2011; de Nicola, 2015; Hill et al., 2013; Mobarak and Rosenzweig, 2012).^3^ Mobarak and Rosenzweig (2012) find that, for every kilometer increase in the perceived distance of a farmer's land from the weather station, the demand for index-based insurance dropped by over 6 percent. Hill et al. (2016) find that doubling the distance to the reference weather station decreases demand by 18 percent. Based on a discrete choice experiment in eastern India, Ward and Makhija (2018) find that, for every 1 percent increase in basis risk, farmers would need to be compensated with a 3–4 percent reduction in the cost of insurance.

In the presence of basis risk the traditional theoretical predictions regarding the relationship between risk aversion and insurance demand also no longer hold, since the product itself is now risky. Instead, demand is initially increasing in risk aversion before decreasing such that, for very risk-averse farmers, purchasing insurance actually makes them *worse* off (Clarke, 2016). Hill et al. (2016), for example, find that demand for index insurance is inverse U-shaped in risk aversion, and others have documented a negative relationship between risk aversion and demand (Giné et al., 2008).

Indeed, across various countries and contexts, uptake of index insurance has been low even when offered at actuarially-favorable rates. In Ghana, Karlan et al. (2014) find a price elasticity of roughly −2.^4^ Cole et al. (2013a,b) estimate a price elasticity of demand between −1.04 and −1.16 in Andhra Pradesh. Other studies find more moderate price elasticities: Hill et al. (2016) estimate the price elasticity of insurance demand to be −0.58, while Mobarak and Rosenzweig (2012) find the price elasticity to be −0.44.

The emerging evidence around many index insurance products is that subsidies are often required – at least in the short run – to stimulate demand (J-PAL et al., 2016). These subsidies can take various forms, but we focus on discounts and rebates. Discounts and rebates primarily differ in the timing with which the benefits are realized, but they can also interact differently with idiosyncratic behavioral preferences and can have different implications for insurers' business models. In typical index insurance contracts the premium is paid by the insured prior to the start of the coverage period for a promise of later payment conditional upon some adverse event being realized. This can cause liquidity constraints, low trust in the insurance provider, and present bias to constrain insurance demand (e.g., Karlan et al., 2014). In this context discounts can be particularly effective and we would expect them to be more effective than rebates. This would be consistent with Epley et al. (2006), who find that people are generally more likely to spend income framed as a gain from a current wealth state (e.g., a discount on the cost of purchase) than income framed as a return to a prior state (e.g., a rebate). Discounts might be especially successful in addressing liquidity constraints in the context of smallholder agriculture, since the decision to purchase insurance is often concurrent with decisions regarding agricultural production (e.g., investments in agricultural inputs). For insurers, providing discounts may result in increased insurance sales, but at the expense of deteriorating revenues relative to value-at-risk, which may constrain their ability to reinsure. Providing subsidies in the form of rebates would ameliorate some of these constraints, but likely at the expense of lower insurance demand.

There is both theoretical and empirical evidence that behavioral preferences may lead some individuals to respond favorably to rebates. In the presence of basis risk the traditional theoretical predictions regarding the relationship between risk aversion and insurance demand also no longer hold, since the product itself is now risky. Instead, demand is initially increasing in risk aversion before decreasing such that, for very risk-averse farmers, purchasing insurance actually makes them worse off (Clarke, 2016; Hill et al., 2016; Giné et al., 2008). Rebates may be particularly attractive when there is significant ambiguity about the payout as a result of basis risk (Serfilippi et al., 2016). Hyperbolic discounting may also lead some individuals to be more influenced by rebates than discounts, as they are a form of forced saving for a future and uncertain period (Ashraf et al., 2006; Ito and Kono, 2010).

In cases where sufficient uptake of insurance has occurred, impacts of index-insurance have largely been positive (Carter et al., 2014). Janzen and Carter (2013) find that index insurance positively affects pastoral farm households in Kenya following a shock: asset-rich households are less likely to engage in distress sales of livestock to smooth consumption, while asset-poor households are less likely to destabilize consumption by reducing meals. Karlan et al. (2014) found that insurance led Ghanaian farmers to increase agricultural expenditures on their farms along both the extensive as well as the intensive margin. Insured farmers cultivated nearly an acre more land and spent nearly 14 percent more on land preparation costs while simultaneously increasing expenditures on modern inputs (mostly fertilizers) by nearly 24 percent. In Burkina Faso, Senegal, and Ethiopia farmers who had weather index insurance purchased more fertilizer (Delavallade et al., 2015; Berhane et al., 2014). In Andhra Pradesh and Tamil Nadu, India, two separate RCTs find that insurance causes farmers to invest in higher-return, rainfall-sensitive cash crops (Cole et al., 2013a,b; Mobarak and Rosenzweig, 2012).

## 3 Study context and experimental design

### 3.1 Context and overall study design

This study took place in Bogra district of Rajshahi Division in northwestern Bangladesh. Bogra is largely rural and livelihoods are heavily dependent upon agriculture, with rice double-cropping the predominant cropping system. While much of Bogra is characterized by alluvial soils fertilized by siltation from floodwaters, much of it is simultaneously drought-prone: farmers in Bogra identified drought and crop diseases as the major sources of crop loss during the monsoon season (Clarke et al., 2015). During the annual monsoon season, in which Bangladesh receives roughly 80 percent of its annual rainfall, there are three distinct types of droughts. Early season droughts arise due to the delayed onset of the annual monsoon and can affect the timing of activities such as transplanting, which in turn affects both the area cultivated and yields. Mid-season droughts typically arise as intermittent, prolonged dry spells and, depending on their timing, reduce crop productivity. Finally, late-season droughts arise due to early monsoon cessation and are particularly damaging for rice production, as they tend to coincide with flowering and grain filling stages in the crop growth cycle.

The study was implemented with the cooperation of a local NGO, Gram Unnayan Karma (GUK), that provides a range of services to households in Bogra, including microfinance, non-formal primary education, primary healthcare, and women's empowerment activities. GUK was established in 1989 and operates primarily through village-level groups consisting of female “members” who voluntarily register to participate and benefit from GUK activities. The study was initiated with a baseline survey in the spring of 2013 and culminated with a follow-up survey 12 months later (see Table A1 in Appendix A).

Three *upazilas* (subdistricts) within Bogra were selected on the basis of proximity to the district meteorological station operated by the Bangladesh Meteorological Department.^5^ Within each of the three selected *upazilas*, 40 villages were randomly selected for inclusion in the study. From within each of these 120 villages, a sample of GUK members (averaging between 15 and 20 members per village) was randomly selected to participate in the study. The baseline survey proceeded in May 2013 among the total sample of 2300 households from these 120 villages. GUK marketed the index insurance product (described in greater detail below in Section 3.2) in half of the sample villages (the randomly-assigned treatment villages) from late May until late June. The coverage period for the insurance policy ran from mid-July to mid-October, as described below. Payouts were made in November 2013 and follow-up surveys were conducted from June to July 2014. All told, attrition proved to be a very minor concern, as virtually all (97 percent) of the households interviewed during the baseline survey were also interviewed during the follow-up survey.^6^

Table 1 presents average characteristics of households in our sample by treatment category with a statistical comparison between the two groups. While there are some differences between households in the treatment and comparison villages along some demographic dimensions, there are no significant differences in terms of the agricultural inputs and outputs that will be considered as outcomes in the subsequent treatment effects regressions. The demographic differences can be controlled for through their inclusion as covariates when we attempt to econometrically identify treatment effects. The overall sample presents the following characteristics on average. Roughly 96 percent of the households are headed by males who, on average, are about 43 years of age. Among these household heads, the number of years of schooling completed averages about 3.5 years. In total, households cultivated roughly 94.2 decimals (0.94 acres) of land on all crops in the 12-month recall period prior to the baseline survey in 2013, including 50 decimals cultivated under *aman* rice and 60 cultivated under *boro* rice. A little over a quarter (30 percent) of our sample owns a savings account with a bank, while on average less than 20 percent of households are members of informal savings groups. A slightly higher proportion of households in treatment villages believe that their cash savings are sufficient to enable them to weather most typical cash flow shortfalls or other livelihood disruptions (40 percent to 30 percent). Nearly all (91 percent) households had taken a loan in the 12-month recall period prior to the baseline survey. These indicate familiarity with financial products and formal institutions, and suggest some basic capacity to understand the insurance product.

**Table 1:** Characteristics of households in randomly allocated treatment and comparison villages.

	Sample	Comparison	Treatment	Difference
**Household characteristics**				
Gender of household head (male = 1)	0.96 (0.00)	0.95 (0.01)	0.97 (0.01)	0.02^∗∗^ (0.01)
Age of household head	42.74 (0.26)	42.56 (0.37)	42.91 (0.38)	0.35 (0.62)
Household size (persons)	4.33 (0.03)	4.26 (0.04)	4.39 (0.04)	0.13^∗^ (0.07)
Education (highest class completed) of household head	3.52 (0.09)	3.37 (0.12)	3.66 (0.13)	0.29 (0.28)
Total land owned and cultivated (decimal)	94.16 (1.98)	94.67 (2.93)	93.67 (2.68)	−1.00 (6.09)
Number of years household has been a member of GUK	3.82 (0.07)	4.08 (0.10)	3.56 (0.10)	−0.53^∗∗^ (0.26)
Household has a savings account with a formal bank (=1)	0.29 (0.01)	0.30 (0.01)	0.28 (0.01)	−0.02 (0.03)
Household cash savings is adequate	0.31 (0.01)	0.26 (0.01)	0.35 (0.02)	0.09^∗∗∗^ (0.03)
Household is a member of an informal savings group (=1)	0.19 (0.01)	0.21 (0.01)	0.17 (0.01)	−0.04 (0.03)
Household asset index (PCA)	0.06 (0.02)	0.01 (0.03)	0.11 (0.03)	0.10 (0.09)
Partial risk aversion coefficient	3.66 (0.07)	3.65 (0.11)	3.68 (0.10)	0.03 (0.18)
Ambiguity averse (=1)	0.71 (0.01)	0.68 (0.01)	0.75 (0.01)	0.07^∗∗^ (0.03)
Time preferences	2.70 (0.08)	2.78 (0.12)	2.62 (0.11)	−0.15 (0.22)
Household trusts GUK management (scale from 1 to 4)	2.94 (0.01)	2.88 (0.02)	2.99 (0.02)	0.11^∗∗^ (0.05)
Distance from *upazila* extension office (km)	10.32 (0.14)	9.63 (0.19)	11.00 (0.19)	1.37 (1.12)
**Monsoon season 2012**				
Total area cultivated during monsoon season (decimals)	64.31 (1.20)	65.67 (1.79)	62.99 (1.59)	−2.68 (3.67)
Total land under *aman* rice (decimals)	49.56 (1.04)	50.03 (1.50)	49.10 (1.44)	−0.93 (3.61)
Total harvest of *aman* rice (kg)	745.24 (17.15)	711.89 (23.91)	777.76 (24.53)	65.86 (62.14)
Total expenditures on fertilizers (BDT)	1929.86 (42.62)	1980.97 (61.34)	1880.03 (59.21)	−100.94 (135.10)
Total expenditures on pesticides (BDT)	256.48 (8.39)	257.79 (11.83)	255.19 (11.91)	−2.60 (32.26)
Total expenditures on hired labor (BDT)	1496.94 (42.10)	1529.32 (62.35)	1465.37 (56.73)	−63.95 (132.61)
Total expenditures on irrigation (BDT)	656.36 (23.06)	612.97 (31.74)	698.67 (33.39)	85.70 (84.33)
Total expenditures on purchased seeds (BDT)	371.67 (11.66)	364.93 (16.47)	378.23 (16.53)	13.30 (31.01)
**Dry season 2012**–**13**				
Total area cultivated during dry season (decimals)	91.95 (1.70)	91.91 (2.44)	91.99 (2.38)	0.08 (6.13)
Total land under *boro* rice (decimals)	60.29 (1.12)	60.26 (1.63)	60.33 (1.54)	0.08 (3.88)
Total harvest of *boro* rice (kg)	1378.51 (26.30)	1348.80 (37.80)	1407.47 (36.59)	58.67 (91.36)
Total expenditures on fertilizers (BDT)	4110.99 (87.90)	4031.75 (124.28)	4188.24 (124.34)	156.49 (284.61)
Total expenditures on pesticides (BDT)	500.44 (14.26)	493.88 (20.16)	506.85 (20.17)	12.97 (51.96)
Total expenditures on hired labor (BDT)	2688.10 (75.58)	2735.90 (110.54)	2641.49 (103.31)	−94.41 (259.13)
Total expenditures on irrigation (BDT)	2894.40 (59.69)	2841.41 (85.33)	2946.08 (83.52)	104.66 (185.85)
Total expenditures on purchased seeds (BDT)	866.94 (27.97)	807.45 (37.86)	924.95 (41.04)	117.50 (91.60)
Number of observations	1983	979	1004	

Note: ^∗^ Significant at 10 percent level; ^∗∗^ Significant at 5 percent level; ^∗∗∗^ Significant at 1 percent level. Figures reported in the fifth column are based on coefficient estimates from linear regressions of the form *x*_*ij*_ = *α* + *βT*_*i*_ + *ɛ*_*ij*_, where *x*_*ij*_ is the characteristic over which balance is being tested (i.e., the variable described in the row header) and *T*_*i*_ is a binary indicator equal to 1 if the household was in a village assigned to the insurance treatment arm. The standard errors from these regressions (in parentheses below the point estimates) have been adjusted for clustering at the village level. Statistical significance of these differences was based on a *t*-test of the estimated coefficient *β* for each characteristic.

Households in our sample have been GUK members for about 4 years, though those who reside in villages randomly allocated to the insurance treatment group have a slightly shorter legacy than those residing in villages randomly allocated to the comparison group (3.6 years vs. 4.1 years). The fact that households in the treatment villages have typically maintained a relationship with the organization providing insurance is important, as it is at least somewhat indicative of their degree of trust in the organization. We also measure trust based on individual farmers' beliefs that GUK management will act in the best interest of their clients. The level of trust in our sample was quite high in general (2.9 on a scale of 1–4, where 4 indicates a high level of trust in GUK management), and slightly higher among treatment households than comparison households. Trust in and familiarity with the insurance provider has been shown to be an important determinant of insurance demand and can have implications for uptake (Karlan et al., 2014; Cole et al., 2014, Cai et al., 2015a; Cai et al., 2015b). Trust in GUK management might be particularly relevant for households that are offered rebates, since they will have to trust that the rebate (in addition to any insurance payouts that might be due) will actually be delivered. On the other hand, the nature of the subsidy (discount vs. rebate) only matters to those with some demand for insurance, which itself is conditional on trust. Therefore if you limit the sample to those with any demand at all for insurance you are limiting it to farmers with at least some degree of trust. In other words, if you trust GUK to make an insurance payout, you likely also trust them to follow through with the rebate. If you do not trust them to make an insurance payout, it no longer matters whether you trust them to give a rebate, since you will most likely not purchase insurance regardless of the nature of the subsidy. The salience of this characteristic may be magnified for households that are risk-averse. Households in the sample show an average level of partial risk aversion of 3.7, which is classified as severe according to Binswanger (1980).

When considering outcome variables of interest, we note there are no systematic differences in households in treatment and comparison villages along agricultural dimensions at baseline. In particular, after controlling for the clustered nature of the intervention, expenditures on agricultural inputs such as irrigation, seed, fertilizers, pesticides, and hired (i.e., non-family) labor during both the 2012 monsoon season and the 2012-13 dry are statistically indistinguishable between treatment and comparison villages. Similarly, the area cultivated, both in total as well as under rice, as well as rice harvests and rice yields are also statistically indistinguishable between treatment and comparison households during both the 2012 monsoon and 2012-13 dry seasons.

### 3.2 The insurance product

The insurance policy covered the monsoon season (July 15 - October 14, inclusive), a period characterized by large amounts of rainfall on average, but also with significant variability.^7^ While the *aman* rice crop is largely rainfed, we note that there is widespread evidence of functioning irrigation markets during this season as well, with groundwater irrigation serving to supplement deficient rainfall.^8^ The insurance design was informed by extensive formative research. In related work, Clarke et al. (2015) conducted an insurance demand-elicitation exercise in Bogra in which farmers demonstrated their interest in various types of insurance products by allocating a monetary endowment across various financial instruments. Clarke and co-authors find that insurance demand varies with the prevalence of the risk that it insures, especially for the case of area yield and dry-days insurance. Based on this formative research, the insurance product developed for the present study protects households against a long period of successive “dry-days” during the monsoon season and against low average area yields as a result of overall rainfall deficiency, pests, crop diseases, or flood.^9^

According to the policy specifications, the insured would receive a payout if a long period of successive dry days was recorded at the local weather station *or* if the average area yield in the *upazila* was very low.^10^ The dry days triggers were established based on 30 years' worth of historical rainfall data from the Bangladesh Meteorological Department. If the longest dry spell that occurred was at least 14 days, the policy would pay out BDT 600.^11^ On average, this type of dry spell occurs roughly once every decade. If the longest dry spell that occurred was 12 or 13 days in length, the policy holder would receive a payment of BDT 300. This type of dry spell occurs roughly once every five years.^12^ Actual rainfall measurements were recorded at the *upazila* agricultural extension offices in each of the three *upazilas*, allowing for potential heterogeneity in rainfall realizations – and thus the performance of the index insurance product – over space. If the dry days triggers were not met the insurance payouts could still be triggered based on the outcome of a crop-cutting exercise undertaken by Bangladesh Bureau of Statistics at the *upazila* level. If the average yield from 30 randomly selected plots from the *upazila* was less than 26 maunds per acre, the policy would pay out BDT 300.^13^ Each policy could pay out a maximum of one time based on the greatest severity of the three events – if any – that occurred.

The base cost per unit of insurance was BDT 100, roughly 10 percent lower than the actuarially fair price. While not explicitly tied to rice production, each policy was meant to cover revenue from 10 decimals (0.1 acres) of land cultivated under transplanted *aman* rice. On average, households in the sample cultivate roughly 50 decimals under *aman* rice during the monsoon season. Households had the option of purchasing multiple units of insurance based on the amount of land they cultivate during the monsoon season, thereby reducing any incentive to view the insurance as a gamble.

### 3.3 Insurance marketing

Informational sessions were held in all treatment villages during which trained product specialists from GUK introduced the insurance product. These training sessions were held about two weeks in advance of the actual sales period. The training sessions typically consisted of 15–20 participating households, including both the female GUK member and her husband or other male family member responsible for decision making. All households that were GUK members within these villages were invited to attend these sessions and were eligible to buy the insurance as long as they cultivated during the monsoon season. A large percentage of invited households (more than 96 percent for each focus group meeting) attended these sessions.

Each training session lasted 3–4 h and was designed to provide information to help farmers make well-informed decisions about whether or not to purchase insurance. Each session covered the nature of risk to agricultural production and the strategies that households could use to cope with these risks. The insurance product that was being offered was described and the possibility of basis risk was discussed. Various hypothetical cases were considered for the purpose of exposition. The session concluded by setting a date and time for the follow-up informational session and discussing how participants could go about purchasing the insurance product, if interested. To simplify the purchasing process, agents distributed insurance demand forms that participants were asked to complete prior to the next appointment.

Since many index insurance programs have suffered from low demand in the past, we were interested in studying the differential effects of alternative incentive mechanisms on stimulating insurance demand. To this end, we randomly allocated half of the villages in the treatment group to receive an instantaneous discount (reduction in the purchase price), while the other half received a rebate (portion of the purchase price refunded at a later date, toward the end of the monsoon season). We further randomized the level of discount or rebate received at the village level with a skewed distribution such that a higher proportion of sample villages were eligible to receive a higher monetary incentive in order to ensure a reasonable demand for the insurance. Table 2 provides the distribution of villages by the level of discount or rebate. Participants were informed at the end of the training session that they would be the recipient of a discount or rebate. The value of the discount (rebate) the village was to receive was randomly selected in the training session. Thus, participants were aware of the effective purchase price for insurance as well as any future refunds they would be entitled to prior to committing to purchase any.

**Table 2:** Distribution of discounts and rebates among treatment villages.

Level of discount/rebate (percent)	Number of villages in treatment group		
	Discount	Rebate	Total
10	1	1	2
20	1	1	2
30	1	1	2
40	1	1	2
45	1	1	2
55	1	1	2
60	2	2	4
65	3	3	6
70	4	4	8
75	5	5	10
80	3	3	6
85	1	1	2
90	6	6	12
Total	30	30	60

In every treatment village, four such information sessions were held to ensure that households were well-informed and in the best position to make the decision to purchase the insurance. Apart from GUK membership, there were no restrictions on who could attend a given information session, so those who had previously attended one session could attend subsequent sessions in order to address any questions or to purchase the insurance. Indeed, given the high participation rates throughout, it is clear that many GUK members attended all of these information sessions.

### 3.4 Weather realizations and index insurance performance

Based on rainfall measurements at the three *upazila* agricultural extension offices, there were severe droughts that occurred in each of the *upazilas* (dry spells exceeding 14 days) during the 2013 monsoon season (Fig. A3 in Appendix A). Despite the *upazilas* being in relatively close proximity, this figure highlights the extent to which rainfall realizations can vary over space during the insurance coverage period, ranging from 616 mm in Bogra Sadar *upazila* to only 317 mm in Sariakandi *upazila*. In Bogra Sadar *upazila*, there was a 16 day dry spell from September 10 through September 25; in Gabtoli *upazila*, there was a 16 day dry spell from September 13 through September 28; in Sariakandi *upazila*, there was a 14 day dry spell from September 12 through September 25. Since these dry spells met or exceeded the upper threshold specified in the insurance contracts, all policyholders were entitled to a BDT 600 payout per unit of insurance purchased. GUK administrators ensured that all payouts to farmers were distributed within one month of the culmination of the insurance coverage period.

## 4 Demand for weather insurance

### 4.1 Empirical approach

**Fig. 1:**
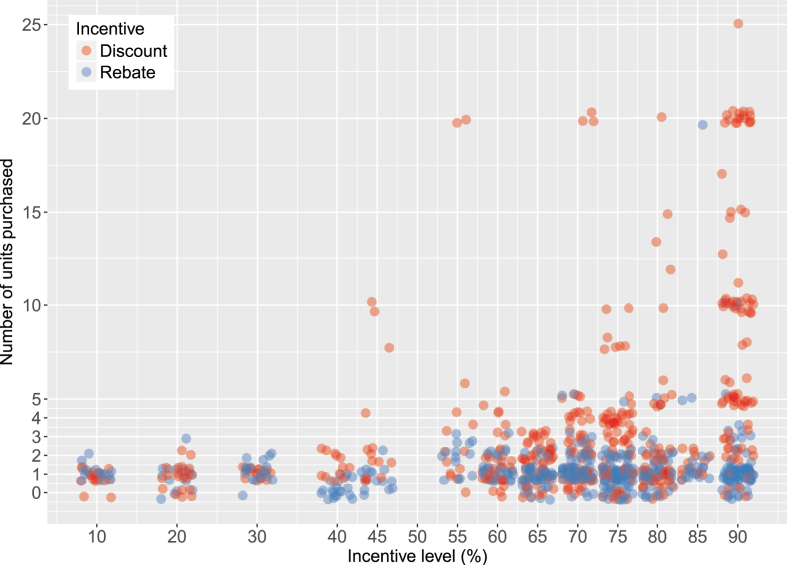
Scatter plot of index insurance purchases by incentive type.

We begin by exploring the determinants of index insurance demand. Fig. 1 illustrates the patterns of insurance take-up at varying levels of discounts and rebates. Here, we focus only on the households from the treatment villages. Our randomization of treatment villages to receiving either a discount or rebate allows us to compare how these two incentives affect households' insurance purchasing decisions, while additional randomization of the level of discount or rebate allows us to assess farmers' sensitivity to the effective cost of insurance, and, ultimately any differential in their price sensitivity depending on the nature of the incentive offered. Since take-up of insurance was very high (87 percent of households in the treatment villages purchased at least one unit of insurance), we focus on how the level and nature of the incentive and other characteristics affect the coverage level (i.e., the number of units) that farmers purchase. Among those farmers that purchased insurance, the average coverage amount was nearly three units purchased, though there was a nontrivial number of households who purchased 10 or more units (up to a maximum of 25 units). To put this into the perspective of coverage area, farmers that purchased insurance on average purchased enough to cover roughly 83 percent of their total area under *aman* rice cultivation.^14^

We begin by estimating the following linear regression model to estimate the impact of discounts and rebates on demand:

(1) $$Q_{i}=\alpha +\beta L_{i}+\theta \left(L_{i}\times R_{i}\right){+\;ɛ}_{i}$$

where *Q*_*i*_ is the number of insurance units purchased by household *i*, *L*_*i*_ is the level of the rebate or discount, *R*_*i*_ is a binary variable indicating whether a household received a rebate (*R*_*i*_ = 1) or a discount (*R*_*i*_ = 0), and *ɛ*_*i*_ is an idiosyncratic error term. This model assumes that the intercepts under discounts and rebates is the same (e.g., *E*[*Q*_*i*_; *R*_*i*_ = 0, *L*_*i*_ = 0] = *E*[*Q*_*i*_; *R*_*i*_ = 1, *L*_*i*_ = 0]), but allows for the slopes of the demand response curves (*β* and *β* + *θ*, respectively) to differ.

So long as *β* > 0 and *β* + *θ* > 0, insurance demand will be increasing in both discounts and rebates. Both theory and empirical evidence would largely support both of these predictions, though there has not yet emerged a consensus as to the relative magnitudes of these incentive response slopes, nor as to whether *θ* > 0. In general, the impact of a discount is expected to be larger than the impact of a rebate (i.e. *θ* < 0 < *β*) on account of present bias, greater liquidity constraints at the beginning of the season than at the end of the season, and the uncertainty that may surround whether or not the rebate will be paid. However, if individuals have discontinuous preferences between certain and uncertain outcomes, this preference for discounts may be moderated (Serfilippi et al., 2016). We may find that the rebate has heterogeneous effects depending on the degree to which individuals are credit constrained, value the present over the future, value certainty or the degree to which they perceive the benefits of the insurance as uncertain.

To assess whether rebates have heterogeneous effects on insurance demand, we estimate the following equation:

(2) $$\begin{array}{c} Q_{i}=\alpha +\beta L_{i}+\theta \left(L_{i}\times R_{i}\right)+\overset{J}{\underset{j=1}{\sum }}\gamma_{j}x_{ij}+\overset{K}{\underset{k=1}{\sum }}\;\left[\xi_{k}\left(z_{ik}\times L_{i}\right)+\phi_{k}\left(z_{ik}\times R_{i}\right)\right]{+\;ɛ}_{i} \end{array}$$

where **x**_*i*_ = 〈*x*_*i*__1_, *x*_*i*__2_, …, *x*_*iJ*_〉 is a vector of household- and farm-level characteristics and **z**_*i*_ ⊂ **x**_*i*_ = 〈*z*_*i*__1_, *z*_*i*__2_, …, *z*_*iK*_〉 is a subset of household-level characteristics (time preferences, risk aversion, and susceptibility to basis risk, proxied by distance to the agricultural extension office) that are used to test for heterogeneous subsidy effects, and *ɛ*_*i*_ is an idiosyncratic error term. The parameter vectors *ξ* = 〈*ξ*_1_, *ξ*_2_, …, *ξ*_*K*_〉 and *ϕ* = 〈*ϕ*_1_, *ϕ*_2_, …, *ϕ*_*K*_〉 also provides some valuable insight into insurance demand, particularly with respect to dimensions of demand heterogeneity.

### 4.2 Results

The results of estimating equations (1), (2) by least squares are shown in Table 3 in columns (1)–(6). Not surprisingly, demand for insurance is price-sensitive, with insurance demand increasing with the level of the associated discount ($\hat{\beta }>0$) or rebate ($\hat{\beta }+\hat{\theta }>0$), and robust to various specifications. Implicitly, these results suggest a price elasticity of insurance demand of −0.65, which is well within the range of other observed elasticity estimates (e.g., Mobarak and Rosenzweig, 2012; Hill et al., 2016; Cole et al., 2013a,b; Karlan et al., 2014). Since $\hat{\theta }<0$, we know that the slope of the demand response to rebates is flatter than the demand response to discounts, though we can reject the null hypothesis that $\hat{\beta }+\hat{\theta }\leq 0$, suggesting that insurance uptake is still increasing in rebate levels despite the preference for discounts.^15^ The statistically insignificant intercept term $\hat{\alpha }$ suggests that demand for this specific insurance would be essentially nil without any sort of incentive to encourage take-up.

**Table 3:** Estimates of insurance demand.

Dependent variable: insurance units purchased (#)	(1)	(2)	(3)	(4)	(5)	(6)
Intercept	−0.829 (0.759)	−1.086 (1.363)	−1.350 (1.415)	−1.517 (1.480)	−3.169^∗^ (1.883)	−3.712^∗^ (1.894)
Level of incentive (BDT)	0.067^∗∗∗^ (0.018)	0.070^∗∗∗^ (0.018)	0.076^∗∗∗^ (0.017)	0.080^∗∗∗^ (0.017)	0.113^∗∗∗^ (0.034)	0.124^∗∗∗^ (0.032)
Level of incentive × rebate binary indicator	−0.038^∗∗∗^ (0.010)	−0.040^∗∗∗^ (0.010)	−0.043^∗∗∗^ (0.010)	−0.046^∗∗∗^ (0.011)	−0.073^∗∗∗^ (0.020)	−0.077^∗∗∗^ (0.020)
Trust in GUK management		0.198 (0.225)	0.199 (0.222)	0.180 (0.230)	0.194 (0.195)	0.188 (0.197)
Time preferences (ann. discount rate)		−0.003 (0.024)	0.032 (0.062)	−0.005 (0.024)	−0.006 (0.023)	0.048 (0.055)
Partial risk aversion coefficient		−0.037 (0.023)	−0.038^∗^ (0.023)	0.023 (0.080)	−0.031 (0.022)	0.070 (0.063)
Ambiguity averse (=1)		0.023 (0.226)	0.012 (0.226)	−0.010 (0.222)	0.092 (0.213)	0.066 (0.211)
Distance to ag. extension office (km)		−0.126^∗∗∗^ (0.046)	−0.127^∗∗∗^ (0.046)	−0.126^∗∗∗^ (0.046)	−0.005 (0.140)	−0.022 (0.139)
Time preferences × rebate			0.105^∗∗∗^ (0.039)			0.041 (0.042)
Time preferences × level of incentive			−0.001 (0.001)			−0.001 (0.001)
Partial risk aversion × rebate				0.126^∗∗∗^ (0.047)		0.061 (0.045)
Partial risk aversion × level of incentive				−0.002 (0.001)		−0.002 (0.001)
Distance to ag. extension office × rebate					0.224^∗∗∗^ (0.084)	0.218^∗∗^ (0.085)
Distance to ag. extension office × level of incentive					−0.003 (0.002)	−0.003 (0.002)
Household/farm controls	No	Yes	Yes	Yes	Yes	Yes
Number of observations	1004	1004	1004	1004	1004	1004
*R*^2^	0.202	0.266	0.269	0.271	0.306	0.309

Note: ^∗^ Significant at 10 percent level; ^∗∗^ Significant at 5 percent level; ^∗∗∗^ Significant at 1 percent level. Standard errors adjusted for clustering at the village level in parentheses. Household/farm controls include household head age, gender, and highest education level, household size, asset holdings (index, constructed by principal components analysis), the length of time the household has been a member of GUK, total land holdings, a binary indicator for whether the household has a savings account at a formal financial institution, a binary indicator for whether the household is a member of an informal savings group, and the household head's perceptions of the sufficiency of cash savings.

In the most parsimonious specification (column [1]), the results suggest that, on average, there would not be any demand for insurance (i.e., at least a single full unit) unless there was at least a 23 percent discount on the actuarially-fair cost of insurance, or a 40 percent rebate.^16^ This is consistent with the oft-cited narrative that farmers would not be willing to purchase any form of crop insurance, even if at actuarially fair prices. In column (2), we control for a series of household and farm-level characteristics that might plausibly influence insurance take-up (e.g., as suggested by existing literature; see e.g Platteau et al., 2017). The demand responses to the incentive mechanisms is largely robust to the inclusion of these other covariates, and their inclusion does not contribute much to explaining the variation in insurance take-up. Columns (3)–(6) introduce a series of interactions that allow us to test for heterogeneous effects of both the level (in percentage terms) and nature of the subsidy (i.e., whether the subsidy took the form of a rebate) on insurance demand. By and large, the inclusion of these interactions only marginally increases our ability to explain the variation in insurance take-up relative to the more parsimonious specifications. Across all specifications, interactions with the level of the subsidy are insignificant, suggesting that the effect of changes in the subsidy level has a fairly uniform effect throughout the population. As was observed in the more parsimonious model, across all models in columns (2)–(6) we find $\hat{\theta }<0$, though we reject the null hypothesis that the linear combination $\hat{\beta }+\hat{\theta }\leq 0$.

These results suggests that farmers generally prefer discounts to rebates. Overall, we observe that farmers being offered a discount on the cost of insurance (averaging 70 percent of the base cost of the insurance policy) purchase roughly 3.5 units of insurance, whereas farmers being offered a rebate (also averaging 70 percent of list price) purchase only about 1.2 units of insurance. For a given incentive level, receiving a rebate instead of a discount results in approximately 57 percent fewer units purchased. The timing of the implicit cost reduction is clearly important in farmers' insurance-purchasing decisions.

In column (3), we examine whether individuals who are more patient reduce demand less when faced with a rebate instead of a discount. Our estimates of the implicit discount rate among the farmers in our sample from survey responses suggest a substantial discounting of future receipts (on average, roughly a 262 percent annual discount rate). We might expect that increasing hyperbolic discount rates would result in a stronger preference for present consumption, which would presumably be higher among those receiving a discount. Interestingly, the results reported here seem to suggest the opposite. Specifically, these results suggest that farmers with a higher discount rate would demand more insurance if they were given a rebate rather than a discount. While this result may not be an empirical regularity, there is a plausible explanation for this result. In attempting to explain why individuals with hyperbolic time preferences would purchase more health insurance than those without such time preferences, Ito and Kono (2010) argue that this phenomenon reflects individuals' use of health insurance as a commitment device that facilitates the ‘prepayment’ of healthcare expenses, given their awareness of their own self-control problems and inability to save to buffer against future healthcare expenditures. In our particular case, it is apparently not only the insurance itself that acts like a commitment savings vehicle, but the rebate also serves as a promise of a future cash inflow that could serve as deferred consumption for those with a strong preference for the present. We note, however, that these effects become insignificant in models in which risk aversion is also accounted for (e.g., column [6]), perhaps suggesting that time preferences and risk preferences may be conflated, or in the least are highly correlated.

In column (4), we assess whether rebates work to counter individuals' risk aversion. We see that more risk-averse farmers purchase fewer units than those less sensitive to risk, an effect that is statistically different from zero at the 5 percent level. We test for the presence of an inverse U-shape relationship between insurance demand and risk aversion as predicted in Clarke (2016), but we do not find any evidence of such nonmonotonicity (not shown in Table 3), perhaps on account of the fact that our sample of farmers exhibits quite high levels of risk aversion causing the average coefficient on risk aversion to be negative or on account of high expected contract non-performance. We do find, however, that when we interact the partial risk aversion coefficient with the rebate dummy, demand is higher for those receiving a rebate relative to those receiving a discount (for a given level of risk aversion and a given incentive amount), and this effect is also statistically different from zero. For those that are risk-averse (and who may be especially sensitive to basis risk), the promise of a rebate may provide assurances that they will have some financial recompense in the future, even if they suffer significant crop losses and are not indemnified by the index insurance product. In a comprehensive model that controls for the full suite of explanatory models, this interaction effect too becomes statistically insignificant (though the main effect remains statistically significant), again likely due to the conflation of risk preferences and time preferences.

Finally, in column (5), we assess whether rebates work to counter individuals' susceptibility to basis risk. We use distance from the weather station (located at the agricultural extension office) as a proxy for basis risk. Basis risk is likely to increase with distance from the extension office, so farmers located further from the office are essentially being offered a product with more uncertain benefits.^17^ In the models reported in columns (2)–(4), the estimate for the effect of distance on demand is negative, suggesting that individuals further away from the *upazila* agricultural extension office would purchase fewer units of insurance. When we introduce an interaction between distance and rebate (column [5]), the interaction effect is positive, while the effect of distance for households receiving a discount (i.e., the main effect) becomes insignificant. This suggests that, while being further away from the *upazila* agricultural extension office might generally reduce index insurance take-up on average, providing a rebate to households further away from the agricultural extension office may increase insurance take-up. The rebate introduces some uncertainty around the cost of insurance, and it may be that this uncertainty reduces the effect of increasing uncertainty around benefits (to the extent that increasing distance represents increasing uncertainty over benefits).

The above evidence of heterogeneous demand under rebates versus discounts should be taken with a degree of caution. These results were predicated on the assumption that the underlying demand response to price is linear, whereas Fig. 1 contains evidence of a non-linear response, at least at very high subsidy levels. Specifically, farmers are not price sensitive at low subsidy levels but become price sensitive at higher levels, particularly with discounts. When subsidies reach 45 percent, demand increases much more rapidly with discounts than rebates. Our results in columns (3), (4), and (5) indicate that those negative effects are pronounced under discounts but offset (or offset and then some) under rebates. This finding is at least in some part driven by the fact the demand is low across the board under rebates.

## 5 Effects of insurance on agricultural intensification

### 5.1 Empirical approach

We now move to estimating the effects of our index insurance product on agricultural intensification (specifically expenditures on inputs such as irrigation, pesticides, fertilizer, hired labor, and purchased seeds) and production (specifically total land cultivated, area under rice cultivation, total rice harvested, and rice yields).^18^ As noted in section 2, Carter et al. (2016) show that insurance increases investment in high-return, risk-increasing inputs. As such we would expect to see farmers increasing their use of fertilizer, pesticides, improved seeds or inputs that increase the scale at which they farm such as land cultivated and labor hired.

The expected impact of insurance on irrigation expenditure is more nuanced. Irrigation is a risk-reducing technology since it provides an alternative method for managing drought risk: farmers can simply “turn on the tap” during prolonged dry spells or when monsoon rainfall is otherwise deficient. At face value, therefore, we would not expect spending on irrigation to increase and had we offered a contract that indemnified farmers on the basis of average local yields alone (and if farmers were not, on average, able to mitigate the impact of weather shocks through irrigation), this may be the case. Farmers in Bogra typically purchase groundwater from a tubewell pump owner, and when faced with successive dry days often choose to wait one or two more days to see if their crops will survive without incurring the cost of turning on the tap. By making payouts on successive dry days as well as realized yields, the insurance contract guaranteed farmers that they would receive a payout to cover the increased cost they faced in irrigating their crop during these dry spells. As such, for the insurance contract provided we would expect spending on irrigation to increase were a dry spell to be experienced.

Although the theory predicts that changes in input use induced by the provision of insurance will increase a producer's overall willingness to take risks and increase average production, it does not guarantee that in any one season production outcomes will be higher. In fact in bad states of the world production outcomes could still be lower as a result of the use of strongly risk-increasing inputs.

As previously described, the insurance was offered immediately prior to the 2013 monsoon season, and the *upazila* agricultural extension offices recorded dry spells lasting at least 14 days in each *upazila*, thereby triggering the insurance payout of BDT 600 per unit of insurance purchased.^19^ These payments were made by early December 2013 – around the time when farmers were planting their dry season crops. The timing of the payouts provided liquidity right around the time that farm households were making investments for the 2013-14 dry season. This suggests that there is perhaps some potential that purchasing insurance could directly affect the subsequent agricultural season despite it being outside of the specified insurance coverage period. We thus also examine the impact of insurance on modern agricultural input use and agricultural production in the irrigated dry season.

In turning to the impacts of agricultural insurance, there are different effects that can be measured, each of which have a specific relevance to policymakers. We begin in presenting the intention-to-treat (ITT) effects (i.e., the effect of being randomly allocated to the group being offered insurance, regardless of whether or not the household actually purchases the insurance). Such effects estimates provide broad insight on the potential economy-wide impacts of a subsidized index insurance program such as this that is introduced at scale. We estimate the ITT effects using the analysis of covariance (ANCOVA) estimator, which has been shown to yield greater statistical power than other treatment effects estimators when the correlation in outcome measures over time is relatively low (e.g., see Frison and Pocock, 1992; McKenzie, 2012; Van Breukelen, 2006), which is typically the case with economic outcomes such as expenditures, particularly those in developing countries (McKenzie, 2012). The estimator can be operationalized using least squares by estimating the regression equation

(3) $$Y_{i1}=\alpha +\beta Y_{i0}+\delta T_{i}+\overset{J}{\underset{j=1}{\sum }}\gamma_{j}x_{ij0}{\;+\;ɛ}_{i}$$

where *Y*_*i*__1_ and *Y*_*i*__0_ are the endline and baseline levels of the outcome of interest, respectively; *T*_*i*_ is the binary treatment indicator; **x**_*i*__0_ = 〈*x*_*i*__10_, *x*_*i*__20_, …, *x*_*iJ*__0_〉 is a vector of covariates to control for baseline imbalance; and *ɛ*_*i*_ is an idiosyncratic error term. The *α*, *β*, *δ*, and *γ* terms are parameters to be estimated. Specifically, *δ* is an estimate of the impact of the insurance treatment on the outcome variable. Because the insurance treatment was administered at the village level, we adjust standard errors for clustering at that level.

Alternatively, policymakers might be interested in the effects of insurance on the subpopulation of farmers who actually purchase insurance. The ITT effects provide a biased estimate for the average treatment effect among treated (ATT) households (i.e., those that actually purchase insurance). Assuming the correlation between purchasing insurance and the various outcomes is positive, ITT effects will be downwardly biased estimates of ATT, with the magnitude of the bias inversely related to the proportion of those randomly assigned to be offered insurance making the decision to actually purchase coverage. Since take-up rates in the present study were so high, reliance on the ITT estimates does not result in significantly attenuated estimates of average treatment effects. However, to arrive at estimates of the average treatment effect on insured households, we next estimate local average treatment effects (LATE) by estimating the regression equation

(4) $$Y_{i1}=\alpha +\beta Y_{i0}+\delta T_{i}^{*}+\overset{J}{\underset{j=1}{\sum }}\gamma_{j}x_{ij0}{\;+\;ɛ}_{i}$$

In this case, the treatment indicator of primary interest, $T_{i}^{*}$ is a binary indicator equal to one if household *i* actually purchased insurance, and zero otherwise. To account for the endogeneity of insurance take-up, we instrument for this treatment indicator with a binary indicator variable capturing random assignment into the treatment group. Assuming the standard LATE conditions are satisfied (Imbens and Angrist, 1994), the LATE estimates are estimates of average treatment effects among the subpopulation of households who would always comply with their assignment.

Finally, policymakers may be interested in the effects on agricultural intensification that could be achieved by increasing insurance coverage. From Table 3, it is clear that subsidies (whether in the form of discounts or rebates) have a positive effect on insurance coverage, so if increasing coverage leads to positive agricultural outcomes, this may be an important avenue by which agricultural policies can effect positive agricultural development. To demonstrate this potential effect, consider the following ‘dose response’ treatment effects regression:

(5) $$Y_{i1}=\alpha +\beta Y_{i0}+\delta Q_{i0}+\overset{J}{\underset{j=1}{\sum }}\gamma_{j}x_{ij0}{\;+\;ɛ}_{i}$$

Now the treatment indicator of interest, *Q*_*i*__0_ is a (quasi-)continuous variable representing the number of insurance units (i.e., the coverage level) purchased by household *i*. As was the case with the binary decision to take up insurance in the LATE regression, this coverage level is endogenous. We account for this endogeneity by instrumenting with the subsidy level (as a percentage reduction in the market price). Using this as an instrument requires that the only pathway through which the subsidy affects agricultural decisions is indirectly through its more direct effect on increasing the coverage amount; in other words, the subsidy does not act as a wealth transfer. Given both the absolute and relative magnitudes of the subsidy – no more than BDT 90 (or a little more than USD 1) and only about 1 percent of total monsoon season agricultural expenditures – this is plausible, especially for members of the treatment group receiving rebates rather than discounts.

### 5.2 Results

Estimated treatment effects for the 2013 monsoon season are reported in Table 4.^20^^,^^21^ Given the high take-up rates, ITT effects are very similar in magnitude to the LATEs. As expected, the effect of actually purchasing insurance (LATE) among the households exposed to the insurance treatment is larger in magnitude than the estimated ITT effects, though because of the efficiency loss from instrumental variables regression relative to least squares, and because the standard errors are adjusted in the LATE estimation for clustering in both the first and second stage regressions, the LATE estimates are less precise than the ITT effects estimates. But since the LATE gives the best estimate for the impact of insurance among those who purchased insurance, which may be of greater policy import, we focus our discussion on the LATEs rather than ITT effects.

**Table 4:** Intention-to-treat effects, local average treatment effects, and dose responses of index insurance on agricultural input use and *aman* rice production (monsoon season).

		Agricultural input expenditures during the monsoon season (BDT)									
		Irrigation	Pesticides	Fertilizer	Hired labor	Purchased seeds	Total	Total area cultivated (decimals)	Area Cultivated with rice (decimals)	Quantity of rice harvested (kg)	Rice yield (kg/decimal)
(1)	Intention to treat effect (ITT)	295.535^∗∗∗^ (80.609)	75.570^∗∗∗^ (29.029)	530.620^∗∗∗^ (152.425)	471.966^∗∗∗^ (180.749)	69.789 (52.087)	1708.115^∗∗∗^ (506.255)	10.830^∗∗∗^ (3.113)	1.422 (2.579)	−19.140 (48.604)	−0.622 (0.666)
	Adjusted *R*^2^	0.255	0.264	0.337	0.336	0.062	0.400	0.525	0.389	0.368	0.039
(2)	Local average treatment effect (LATE)	338.574^∗∗∗^ (92.038)	86.556^∗∗^ (33.724)	608.097^∗∗∗^ (177.357)	540.662^∗∗^ (210.619)	79.926 (60.332)	1955.604^∗∗∗^ (590.548)	12.406^∗∗∗^ (3.649)	1.629 (2.954)	−21.868 (55.529)	−0.712 (0.762)
	Adjusted *R*^2^	0.252	0.256	0.327	0.332	0.059	0.393	0.519	0.389	0.369	0.040
(3)	Dose response effect	86.137^∗∗^ (34.711)	31.644^∗∗^ (12.579)	225.736^∗∗∗^ (67.669)	179.196^∗∗^ (78.691)	27.567 (20.058)	658.429^∗∗∗^ (229.060)	4.259^∗∗∗^ (1.413)	0.803 (0.970)	−1.217 (17.543)	−0.140 (0.254)
	Adjusted *R*^2^	0.218	0.230	0.295	0.303	0.053	0.357	0.326	0.385	0.368	0.039
Observations		1977	1977	1977	1977	1977	1977	1977	1977	1977	1977
Mean for comparison group at endline		866.404	295.638	2270.487	2217.143	364.788	7516.416	65.728	44.297	756.047	13.622
Mean for treatment group at endline		1180.275	370.867	2789.237	2661.691	448.905	9233.211	75.532	44.841	741.686	13.144
Unadjusted ITT effect		317.944^∗∗∗^ (106.886)	76.794^∗^ (40.169)	520.995^∗∗^ (210.316)	449.727^∗^ (252.267)	87.287 (57.437)	1737.7798^∗∗^ (714.245)	9.891^∗∗^ (4.753)	0.522 (3.588)	−14.983 (67.290)	−0.482 (0.728)

Note: ^∗^ Significant at 10 percent level; ^∗∗^ Significant at 5 percent level; ^∗∗∗^ Significant at 1 percent level. In LATE regression, binary variable indicating random assignment to the treatment group serves as an instrument for insurance take-up. In dose response regression, the level of the incentive serves as an instrument for the insurance coverage amount. Standard errors adjusted for clustering at the village level in parentheses. For LATE and dose response regressions, standard errors have been adjusted for clustering at the village level in both the first and second stages. ITT, LATE, and dose response regressions control for the baseline level of the outcome variable as well as household and agricultural characteristics for which there was an imbalance at baseline between treatment and comparison groups. Unadjusted ITT regressions report mean differences in levels of outcomes between treatment and comparison at endline without controlling for baseline levels of outcome variables or characteristics for which there were imbalances at baseline.

Focusing first on the risk mitigation effects during the monsoon season, we find that farmers from the treatment group that purchased insurance spent roughly BDT 2000 more on agricultural inputs than did farmers in the comparison group, representing a nearly 30 percent increase over comparison farmers. The increase in input expenditures is not, however, distributed evenly over all inputs. In particular, there is no effect on purchases of seeds, likely reflecting farmers' reliance on recycled seeds of many crops grown during the monsoon season, including *aman* rice. We find that, on average, purchasing insurance results in a roughly BDT 610 increase in fertilizer expenditures (an almost 30 percent increase over comparison farmers), a BDT 340 increase in irrigation expenditures (a nearly 40 percent increase over comparison farmers), a BDT 90 increase in pesticide expenditures (an almost 30 percent increase over comparison farmers), and a BDT 540 increase in expenditures for hired labor (an almost 20 percent increase over comparison farmers).

The increased use of fertilizers is consistent with theoretical predictions that index insurance induces investments in higher-risk, higher-returning activities. Fertilizer has the potential to substantially increase yields, but because fertilizer is expensive and there is the potential for significant crop losses under adverse conditions, farmers are often reluctant to apply chemical fertilizers in an environment of unmanaged risk. This finding that insurance increases fertilizer application (or, more accurately, expenditures on fertilizers) is consistent with other research, both theoretical as well as empirical (e.g., Karlan et al., 2014).

The increase in irrigation costs is also consistent with theory given that the insurance contract offered protection against this cost of production when many successive dry days were experienced, as was the case in the 2013 monsoon season. This result highlights how insurance provided to mitigate the costs associated with managing shocks can encourage households to take appropriate actions to reduce the impact of weather shocks on income. On average, for farmers purchasing irrigation on a variable cost basis, having insurance incentivizes them to undertake approximately one additional irrigation operation, likely to mitigate the effects of the prolonged dry spells.

Similarly, the increase in pesticide expenditures is also consistent with theoretical predictions, especially since the primary risk that farmers face and that the insurance addresses is poor rainfall and not pests. Pesticides, therefore, are risky inputs to the extent that the marginal product of pesticides is higher in good states of the world (that is, in good rainfall conditions) than in bad states (that is, during prolonged dry spells). This point is demonstrated in the theoretical appendix in Karlan et al. (2014).

We do not observe any significant effects on area under rice cultivation, total rice production, or rice yields, despite the increased expenditures on inputs like irrigation, fertilizers, and pesticides that one might otherwise expect to produce yield-enhancing (or at least yield-stabilizing) benefits. There are several possible reasons why we do not observe an effect on yields. The simplest possible explanation is that the increased expenditures that were observed were not utilized on the rice crop. Interestingly, although there is a negligible effect on area cultivated under *aman* rice, there is strong evidence of an expansion in total area under cultivation (roughly 10 decimals, representing an increase on the order of 20 percent relative to comparison farmers).^22^ We are unable to ascertain whether the additional fertilizers and irrigation were used on non-rice crops, since we do not have crop-wise information on input expenditures, but this certainly seems a plausible explanation. Even under the unlikely scenario that the increased input expenditures were intended for rice cultivation – which remains the primary agricultural activity in terms of area, despite the expansion into non-rice production – we note that increased spending on productivity-enhancing inputs does not guarantee higher yields in every state of nature, only presumably higher yields on average. There are many idiosyncratic sources of variation affecting yields – including, but not limited to – genotype × environment interactions that we are unable to control for. As previously noted, there were prolonged dry spells that occurred in each of the *upazilas* during the 2013 monsoon season, and these dry spells all occurred in mid- to late-September, during which time many longer-duration *aman* rice varieties would be reaching their reproductive stages. While the observed increases in irrigation expenditures would likely ameliorate some of the effects of deficient rainfall during this time, since we do not have sufficient information on the timings of these various input applications or irrigation operations, we cannot definitively trace out a causal pathway.

In terms of the effects of an additional unit of insurance amongst the subpopulation of farmers who purchased insurance (i.e., the dose response), we find an increase in overall agricultural expenditures of over BDT 660. Interestingly, this effect on total agricultural input expenditures is of a similar magnitude to the maximum possible insurance payout per unit of insurance (BDT 600), perhaps reflecting farmers' willingness to invest this amount in agricultural inputs with the knowledge that they would most likely be compensated for these expenditures in adverse states of the world, and this effect is not diminished by being increasingly susceptible to basis risk. Notably, the increase in total agricultural expenditures is considerably higher than the payout that farmers should expect to receive per unit on a purely actuarial basis, based on the probabilities associated with events triggering the possible insurance payouts.^23^

Table 5 reports the treatment effects estimates for the dry season.^24^ GUK did not offer insurance to farmers during the dry season, nor are we aware of any other providers of agricultural insurance in the sample area, so all impacts estimated for the dry season are presumably the result of being offered insurance in the monsoon season. As with impacts during the monsoon season, we find that insured farmers spent significantly more on agricultural inputs for dry season production than those in the comparison group. Purchasing insurance increases average input expenditures by roughly BDT 1830 (16 percent) more than farmers in the comparison group. Crop area expanded during the dry season, though the evidence suggests this increase was all dedicated to *boro* rice production. Indeed, the increase in area under *boro* rice is greater than the increase in total area under dry season cultivation, signifying a likely reduction in non-rice cropped area. Again, we do not have data on crop-wise input expenditures, but based on this fact alone it thus seems plausible to assume that any changes in input expenditures can be attributed to investments in *boro* rice production.

**Table 5:** Intention-to-treat effects, local average treatment effects, and dose responses of index insurance on agricultural input use and *boro* rice production (dry season).

		Agricultural input expenditures during the dry season (BDT)									
		Irrigation	Pesticides	Fertilizer	Hired labor	Purchased seeds	Total	Total area cultivated (decimals)	Area cultivated with rice (decimals)	Quantity of rice harvested (kg)	Rice yield (kg/decimal)
(1)	Intention to treat effect (ITT)	271.554^∗^ (147.515)	41.884^∗^ (23.595)	476.264^∗∗∗^ (157.950)	574.082^∗∗∗^ (189.754)	50.461 (37.723)	1601.548^∗∗∗^ (513.951)	5.684^∗∗^ (2.778)	6.812^∗∗∗^ (1.855)	152.673^∗∗∗^ (52.642)	1.133^∗∗^ (0.481)
	Adjusted *R*^2^	0.325	0.299	0.411	0.407	0.124	0.488	0.550	0.583	0.551	0.094
(2)	Local average treatment effect (LATE)	311.347^∗^ (166.835)	48.008^∗^ (27.054)	545.627^∗∗∗^ (176.866)	658.487^∗∗∗^ (218.378)	57.780 (43.103)	1834.814^∗∗∗^ (578.880)	6.515^∗∗^ (3.164)	7.805^∗∗∗^ (2.114)	175.042^∗∗∗^ (60.286)	1.298^∗∗^ (0.553)
	Adjusted *R*^2^	0.329	0.299	0.414	0.407	0.125	0.490	0.551	0.582	0.550	0.093
(3)	Dose response effect	106.024^∗^ (58.872)	18.746^∗∗^ (8.740)	175.587^∗∗∗^ (58.480)	232.487^∗∗∗^ (88.835)	16.422 (14.060)	605.430^∗∗∗^ (211.759)	1.724 (1.052)	2.566^∗∗∗^ (0.822)	61.555^∗∗∗^ (23.521)	0.505^∗∗∗^ (0.193)
	Adjusted *R*^2^	0.322	0.295	0.404	0.382	0.120	0.475	0.546	0.566	0.535	0.073
Observations		1977	1977	1977	1977	1977	1977	1977	1977	1977	1977
Mean for comparison group at endline		2767.521	378.510	3194.675	3149.427	380.353	11550.275	66.941	55.976	1298.257	20.691
Mean for treatment group at endline		3077.057	425.301	3725.353	3751.655	435.363	13348.014	73.179	63.244	1496.468	22.231
Unadjusted ITT effect		314.054 (198.981)	46.869 (35.392)	531.989^∗∗∗^ (204.552)	608.659^∗∗^ (294.434)	56.242 (38.810)	1813.429^∗∗^ (756.991)	6.298 (4.084)	7.320^∗∗^ (3.662)	200.612^∗∗^ (95.224)	1.564^∗∗^ (0.609)

Note: ^∗^ Significant at 10 percent level; ^∗∗^ Significant at 5 percent level; ^∗∗∗^ Significant at 1 percent level. In LATE regression, binary variable indicating random assignment to the treatment group serves as an instrument for insurance take-up. In dose response regression, the level of the incentive serves as an instrument for the insurance coverage amount. Standard errors adjusted for clustering at the village level in parentheses. For LATE and dose response regressions, standard errors have been adjusted for clustering at the village level in both the first and second stages. ITT, LATE, and dose response regressions control for the baseline level of the outcome variable as well as household and agricultural characteristics for which there was an imbalance at baseline between treatment and comparison groups. Unadjusted ITT regressions report mean differences in levels of outcomes between treatment and comparison at endline without controlling for baseline levels of outcome variables or characteristics for which there were imbalances at baseline.

As before, the increased expenditures on inputs for *boro* production are not spread uniformly over the different inputs. For the *boro* crop, we find that insurance led to increases in irrigation, pesticide, fertilizer, and labor expenditures, on the order of roughly BDT 311, 48, 546, and 658 respectively, representing increases over the comparison group of 11, 13, 17, and 21 percent.

Since dry season risk remains uninsured, we cannot attribute these effects to risk management effects. However, because the insurance payments were made following the *aman* rice harvest and just prior to the initiation of the dry season, the payouts generate an income effect. Since we do not have data on how insured farmers' might have behaved with respect to their *boro* input expenditures in the absence of an insurance payout (which, consequently, means in the absence of a measured drought or crop loss during the monsoon season), we cannot say for certain that this effect would only hold after receipt of an insurance payout. If indeed the increased input expenditures during the dry season arose due to receipt of the insurance payout, then perhaps there would be no reason to expect this sort of response in the absence of an insurance payout, especially since there was not a discernible effect on *aman* rice production in Table 4.^25^

In addition to the expansion of *boro* rice area, there were also positive effects on both the rice harvest and rice yields. The increase in *boro* rice area suggests an increase in rice production along the extensive margin, and this raises the question as to whether the increase in input expenditures described above are simply an artifact of an increased area under *boro* cultivation, rather than investments in more intensive *boro* rice production. To test whether farmers also increased input use on the intensive margin, we estimate treatment effects on input use per decimal cultivated (Table 6). We find statistically significant increases in intensive use of fertilizer as well as in total input expenditures per unit of land, and a marginally significant increase in hired labor. Estimates for intensification of irrigation use and purchased seeds also have the expected positive sign despite being statistically insignificant, whereas there is no evidence of more intensive use of pesticides.

**Table 6:** Intention-to-treat effects, local average treatment effects, and dose responses of index insurance on agricultural input use per unit of area (dry season).

		Irrigation	Pesticides	Fertilizer	Purchased		
					Hired labor	seeds	Total
(1)	Intention to treat effect (ITT)	0.729 (2.067)	−0.115 (0.342)	2.859^∗^ (1.622)	4.032^∗^ (2.225)	0.498 (0.490)	9.738^∗∗^ (4.420)
	Adjusted *R*^2^	0.109	0.017	0.012	0.040	0.011	0.023
(2)	Local average treatment effect (LATE)	0.833 (2.357)	−0.131 (0.391)	3.261^∗^ (1.826)	4.600^∗^ (2.560)	0.568 (0.560)	11.110^∗∗^ (4.994)
	Adjusted *R*^2^	0.110	0.017	0.014	0.038	0.010	0.025
(3)	Dose response effect	0.328 (0.783)	0.049 (0.118)	1.229^∗∗^ (0.536)	1.894^∗∗^ (0.947)	0.149 (0.177)	4.192^∗∗^ (1.639)
	Adjusted *R*^2^	0.109	0.017	0.012	0.014	0.009	0.010
Observations		1977	1977	1977	1977	1977	1977
Mean for comparison group at endline		45.498	6.002	50.722	44.501	5.996	177.459
Mean for treatment group at endline		46.394	5.895	53.554	48.688	6.557	187.286
Unadjusted ITT effect		0.943 (2.793)	−0.104 (0.361)	2.834^∗^ (1.691)	4.276^∗∗^ (2.344)	0.573 (0.495)	9.993^∗∗^ (4.688)

Note: ^∗^ Significant at 10 percent level; ^∗∗^ Significant at 5 percent level; ^∗∗∗^ Significant at 1 percent level. In LATE regression, binary variable indicating random assignment to the treatment group serves as an instrument for insurance take-up. In dose response regression, the level of the incentive serves as an instrument for the insurance coverage amount. Standard errors adjusted for clustering at the village level in parentheses. For LATE and dose response regressions, standard errors have been adjusted for clustering at the village level in both the first and second stages. ITT, LATE, and dose response regressions control for the baseline level of the outcome variable as well as farm size (total area cultivated during during monsoon season at baseline) and household and agricultural characteristics for which there was an imbalance between treatment and comparison groups at baseline. Unadjusted ITT regressions report mean differences in levels of outcomes between treatment and comparison at endline without controlling for baseline levels of outcome variables or characteristics for which there were imbalances at baseline.

To convert the treatment effect on rice production to an effect on revenue, we use producer price in Bangladesh at the time of harvest, BDT 14.52 per kg (Food and Agricultural Organization of the United Nations, 2018), as we do not have survey data on rice prices. Using our ITT estimate for the treatment effect on rice production, this amounts to BDT 2217. The corresponding treatment effect on expenditures is BDT 1602, meaning that the rate of return to additional investment was 38 percent. Even if producer prices were substantially lower than the national average (as low as BDT 10.49 per kg), the increased investment would have still been profitable. There are several important caveats to mention regarding this profitability finding. We do not include family labor here, nor do we consider what the alternative use for additional land planted in rice would have been. Both of these commissions could inflate our profitability calculations. However, we include additional investment in all inputs in our calculations but only additional rice production, which could depress these findings downward. Finally, agricultural production is inherently stochastic, depending on many factors besides input use and outside of the farmer's control. Thus, treatment effects could vary greatly on a year to year basis.

Considering again the effects of incremental insurance coverage, we find that each additional unit of insurance encouraged farmers to invest roughly BDT 610 more on agricultural inputs. As was observed in regards to the per unit increase in total expenditures during the monsoon season, the per unit increase in total expenditures during the dry season is of a similar magnitude to the per unit insurance payout that insured farmers received. This suggests that farmers did not simply view the insurance payouts as compensation for the increased monsoon season expenditures that evidently did not yield any visible returns (at least not on the *aman* rice crop), but rather decided to funnel those payouts right back into modern agricultural inputs during the subsequent *boro* rice production. It is encouraging that the total increase in input expenditures across the two seasons exceeds the per unit insurance payout received, and this analysis only focuses on agricultural outcomes, abstracting from other livelihood outcomes that might also emerge from these *ex post* income effects. While we lack the data to properly trace farmers' mental accounting of these risk management effects, income effects, and increased expenditures, these results provide promising evidence for the role of insurance in facilitating the modernization of agriculture.

Until now we have presented results for the pooled treatment group; that is, the group consisting of both households receiving discounts as well as households receiving rebates. This was predicated on the realization that uptake rates were fairly similar between the two groups (90 percent in the discount group and 84 percent in the rebate group) and the assumption that the transfers were not significant enough in absolute terms to constitute a wealth transfer. In order to test whether the small difference in uptake rates and coverage levels between the treatment groups could lead to different results we re-ran the regressions in equations (3), (4), (5) restricting the treatment to those households receiving discounts or rebates. Tables C1–C3 in Appendix C report the results of these regressions (with Panel (A) restricting treatment to only households receiving discounts and Panel (B) restricting treatment to only households receiving rebates) and show no significant difference between the impact of discount and rebate treatments. The one exception is a higher dose response in the dry season recorded among households receiving the rebate treatment. Total expenditures per unit of insurance was higher among households receiving the rebate in the dry season driven by higher spending on farm labor. So while rebates generated lower insurance demand, each unit of insurance purchased generated a greater impact on input expenditure in the subsequent season once the rebate had been received.

## 6 Concluding remarks

The pilot provided treated farmers with easily verifiable and transparent insurance coverage against specified dry spells or low average yields during the monsoon season. Our empirical analysis focuses on both the determinants of insurance demand as well as the subsequent effects of insurance on agricultural practices and production. Our results provide valuable insight into the potential viability of insurance markets, as well as the benefits that such an insurance product might provide, both in terms of risk management and increased income.

Our results on insurance demand are consistent with much of the empirical literature demonstrating that demand for insurance is very price-sensitive. In the absence of financial incentives such as discounts or rebates, our results suggest there would be essentially no demand for our insurance product, even at actuarially-favorable prices. The nature of the incentive also plays a role in stimulating demand. Up-front discounts on the cost of insurance are much more successful at stimulating insurance take-up compared to rebates, which necessarily involve a delay in the receipt of the monetary inducement. This not only affects whether individuals decide to purchase insurance, but also the coverage level that they purchase. On average, individuals receiving a discount purchase roughly 3.5 units of insurance, while those offered a rebate purchase only 1.2 units of insurance.

In our analysis on the impacts of insurance on agricultural intensification and rice production, we find evidence of both *ex ante* as well as *ex post* impacts. The *ex ante* impacts, which we consider as pure risk management effects, translate into significantly higher expenditures on agricultural inputs during the monsoon season, as well as an expansion in the total area cultivated, with this expansion primarily leading to increased cultivation of non-rice crops. Specifically, we find that insurance leads to significantly higher expenditures on fertilizer, hired labor, irrigation, and pesticides. The results highlight that appropriately designed insurance contracts can encourage investments in riskier – though also perhaps more profitable – production as well and helping to mitigate the costs of coping with a weather shock.

During the subsequent dry season, those that had been offered insurance in the monsoon season increased expenditures on irrigation, hired labor, and fertilizer, while also expanding their *boro* rice production. The results support the notion that farmers who were insured during the monsoon season – and thus received an insurance payout – increased their production in the subsequent *boro* season. Since the insurance contract was designed to manage only monsoon season risks, these impacts cannot be considered as arising from a risk management effect. Rather, due to the timing of the insurance payouts (following the *aman* rice harvest and prior to *boro* rice land preparation), these *ex post* effects reflect increased income or liquidity, in this case most directly as a result of the insurance payout. Given insufficient exogenous variation in insurance payout receipts (since all insured farmers receive a payout), we are unable to say with any degree of certainty that this effect would only be present following an insurance payout. This causal pathway seems plausible, though we also suggest that such an income effect could occur even in the absence of an insurance payout, for example due to increased farm profits from *aman* rice production. Parsing out this effect remains a task for future research.

The results highlighted here come from a single study spanning only two agricultural seasons. Furthermore, these results might be compelling largely due to the very high take-up rates, which were induced by unsustainably high incentives on favorably-priced index insurance. It remains to be seen whether such an index insurance program can be sustained under alternative conditions, whether positive experiences with insurance programs can stimulate future demand even without incentives, or, ultimately, whether the *ex ante* and *ex post* impacts of insurance would be realized without the sizable incentives or in the absence of insurance payouts. The large number of related studies that are ongoing in other countries should provide more insight into these unanswered questions.

## Footnotes

1 Transplanted *aman* rice is the most common crop grown during the annual monsoon, typically transplanted from late June to August, and harvested in November or December.

2 *Boro* rice is the most common crop grown during the dry season following the monsoon, typically transplanted from December through February, and harvested in April or May.

3 While basis risk is commonly conceptualized as the mismatch between weather conditions on farmers' fields and those at the weather station or other site at which the weather variables constructing the index are measured, basis risk more broadly refers to any genesis of insurance contract non-performance, which, in this case, refers to any farm losses not compensated for.

4 This study is a prominent counterexample to the widely observed phenomenon of low demand. At the actuarially fair price, 40 to 50 percent of the farmers in their sample demanded insurance, and on average purchased coverage for more than 60 percent of their cultivated area. The price elasticity is estimated as the mid-point of the arc elasticity between the actuarially-fair insurance and the market price insurance.

5 To get a better sense of the geographic location of the study area, see Fig. A1 in Appendix A.

6 While the initial sample consisted of 2300 agricultural households, with very little attrition between baseline and follow-up, the sample sizes that emerge in Table 1, Table 3, Table 4, Table 5 are smaller than the original sample because we focus on those households that cultivated during both the monsoon season and dry season at baseline.

7 See, for example, the average annual distribution of rainfall depicted in Fig. A2 in Appendix A.

8 Results from a telephone survey conducted prior to the follow-up household survey among a randomly selected sample of farmers in treatment villages indicate that roughly 90 percent of farmers access groundwater to supplement rainfall, with the vast majority of those accessing water resourced through a contractual relationship with a local pump owner (less than 10 percent of those interviewed owned their own pump). The nature of the contracts was widely variable, with most farmers paying a fixed amount (either in cash or as a share of their harvest) at the end of the season. Roughly 30–35 percent of those interviewed through this telephone survey reported paying for irrigation on a variable basis, with nearly 2/3 of those paying cash after each operation. Among those paying on a variable basis, most paid roughly BDT 10 per decimal when they irrigated, regardless of the depth or the amount of diesel or volume of water used. When we attempted to implement a similar series of questions during the follow-up household interviews, the responses were somewhat contradictory, with nearly 95 percent of farmers indicating that they utilized a fixed contract for irrigation, although because of the wording of this question it is not clear they understood the difference between fixed and variable contracts.

9 For the purposes of this index insurance product, a “dry-day” was any day (midnight to midnight) in which the cumulative rainfall was less than 2 mm.

10 Table A2 in Appendix A describes these events and how they relate to policy triggers and corresponding payouts.

11 BDT = Bangladeshi taka. At the time of the intervention, the exchange rate was approximately BDT 76 per USD.

12 The return periods for these triggers are based on the assumption that the annual maximum dry spell is distributed according to a Generalized Extreme Value distribution. The location, shape, and scale parameters of this distribution were estimated using maximum likelihood and then used to predict the levels (i.e., dry spell lengths) associated with the corresponding return periods (that is, the inverse of the probability that a particular event will occur in any given year.

13 A maund is a unit of mass commonly used in much of South Asia, roughly equivalent to 40 kg.

14 The covered area is computed by taking the number of units purchased by insured households and multiplying it by 10, since each unit of insurance was essentially meant to cover the revenue from cultivating 10 decimals. We then arrived at the household coverage rate by dividing each insured household's covered area by their area under *aman* cultivation. The figure reported here is simply the arithmetic mean among insured households, though there is significant variation, ranging from 4 to 1500 percent of *aman*-cultivated area.

15 This latter assertion is based on a general test of the linear combination of regression coefficients against a one-sided alternative, with test statistic given by $t=\left(\hat{\beta }+\hat{\theta }\right)/\text{se}\left(\hat{\beta }+\hat{\theta }\right)$, which is distributed according to a *t* distribution with *n* − *k* − 1 degrees of freedom, where *n* and *k* are, respectively, the row and column dimensions of the design matrix. While the sum of the coefficients is small in absolute terms (indicating a fairly flat demand response curve), it is statistically greater than zero, indicating a positive demand response with increasing rebates.

16 This assumes that the intercept on the demand response curves for both discounts and rebates is zero, as implied by the statistically insignificant intercept term in column (1), and is calculated as $L_{i}^{*}=1/\left(\hat{\beta }+\hat{\theta }R_{i}\right)$ evaluated both at *R*_*i*_ = 0 (discount) and *R*_*i*_ = 1 (rebate) and adjusted for the difference between the base price at which the insurance was marketed and the actuarially-fair cost.

17 We note that other factors that could influence demand for insurance are also likely to vary with distance to the upazila agricultural extension office, such as access to input and output markets (since the agricultural extension office is typically located in moderately sized towns that act as market centers), as well as access to farming advice from the extension office itself. It could also be the case that selling insurance through agricultural extension offices could also lead to a negative relationship between distance and demand, as increasing distance would imply higher transaction costs for insured farmers. In the case of the present study, policies were sold during information sessions in each treatment village, so there are no additional costs incurred in purchasing insurance.

18 In reality, land is a factor of production, and thus decisions about the area of land to be cultivated could reasonably be included in a discussion about agricultural input use. In what follows, we differentiate land from the other inputs insomuch as decisions about land involve changes in production along the extensive margin, while decisions about other agricultural inputs typically entail changes in production along the intensive margin.

19 While the insurance itself was not tied to actual on-farm production, each unit of insurance was meant to provide insurance coverage for an area up to 10 decimals (0.1 acres).

20 In the regressions summarized below, we treat total agricultural expenditures and expenditures on irrigation, pesticides, fertilizer, labor, and seeds as independent outcomes, with each independent outcome associated with a unique hypothesis test. In reality, since agricultural inputs are often complementary, our estimation strategy could permit free correlation in error terms across expenditure impact regressions. This could be accomplished by estimating the expenditure impact regressions simultaneously as a ‘seemingly unrelated regression’ (SUR). While not reported here, we have indeed estimated such relationships, and due to the positive correlations between error terms among these different expenditure categories (e.g., due to the complementary nature of many agricultural inputs), we have found both larger and more statistically significant impacts in both monsoon and dry seasons. The estimated effects reported here should, therefore, be treated as conservative estimates of the impact of insurance on input expenditures.

21 For the LATE and dose response regressions in Table 4, the first-stage results are presented in Appendix B, in Tables B1 and B2, respectively. In both sets of first stage regressions, the excluded instruments (random assignment and incentive level, respectively) are highly statistically significant, though the overall regression fit is considerably stronger for the LATE regressions, with the full set of instruments explaining nearly 80 percent of the variation in the endogenous binary insurance take-up indicator.

22 When we attempt to isolate how this area expansion is realized (not reported here), we are only able to explain very little of the overall change in area under different crops during the monsoon season, which suggests that the vast majority of the expansion in cultivated area is very idiosyncratic rather than systematic.

23 In very simple terms, based on a 20 percent probability of a 12–13 day dry spell and a 10 percent probability of a dry-spell lasting at least 14 days, farmers should expect to receive only about BDT 120.

24 The first stage regressions for the LATE and dose response regressions are reported in Appendix B, in Tables B3 and B4, respectively. As was the case with the LATE and dose response regressions for the monsoon season, the excluded instruments (random assignment and incentive level, respectively) are highly statistically significant, though again, the full set of instruments does a much better job of explaining variation in the endogenous binary insurance take-up indicator than in explaining variation in the number of units purchased.

25 It is possible that risk management during the monsoon season might also produce an income effect that results in increased input expenditures during the dry season, regardless of whether an insurance payout was received. Admittedly we do not have an adequate counterfactual at our disposal with which to test this hypothesis, so this remains largely conjectural. Suppose there was *not* a drought during the monsoon season and, consequently, no insurance payout. Because we observe higher input expenditures among insured farmers (vis-á-vis farmers in the comparison group) as a result of risk management and independent of the resultant state of nature, and because we might expect nonnegative marginal productivities for all inputs during *aman* rice production under such conditions, total *aman* rice output for insured farmers should exceed that of uninsured farmers. This, in turn, could produce a similar income effect as the insurance payment and induce increased expenditures on fertilizers during the dry season. The relative magnitudes are impossible to quantify in the absence of a proper counterfactual, but it seems at least plausible that the income effects and increased input expenditures during the dry season could at least indirectly result from the risk management effects that arise during the monsoon season.

## References

[bib1] Alderman H., Haque T. (2007). Insurance against Covariate Shocks.

[bib2] Ashraf N., Karlan D., Yin W. (2006). Tying Odysseus to the mast: evidence from a commitment savings product in the Philippines. Q. J. Econ..

[bib3] Barnett B.J., Mahul O. (2007). Weather index insurance for agriculture and rural areas in lower-income countries. Am. J. Agric. Econ..

[bib4] Barnett B.J., Barrett C.B., Skees J.R. (2008). Poverty traps and index-based risk transfer products. World Dev..

[bib5] Berhane G., Hill R.V., Dercon S., Seyoum Tafesse A. (2014). The Impact of Insurance on Agricultural Investment: Evidence from Ethiopia.

[bib6] Binswanger H.P. (1980). Attitudes toward risk: experimental measurement in rural India. Am. J. Agric. Econ..

[bib7] Binswanger-Mkhize H.P. (2012). Is there too much hype about index-based agricultural insurance?. J. Dev. Stud..

[bib8] Cai J. (2016). The impact of insurance provision on households' production and financial decisions. Am. Econ. J. Econ. Pol..

[bib9] Cai H., Chen Y., Fang H., Zhou L.-A. (2015). The effect of microinsurance on economic activities: evidence from a randomized field experiment. Rev. Econ. Stat..

[bib10] Cai J., de Janvry A., Sadoulet E. (2015). Social networks and the decision to insure. Am. Econ. J. Appl. Econ..

[bib11] Carranco N.M. (2017). Agricultural insurance in Ecuador: evidence of asymmetric information. J. Acc. Taxation.

[bib12] Carter M.R., de Janvry A., Sadoulet E., Sarris A. (2014). Index-based Weather Insurance for Developing Countries: a Review of Evidence and a Set of Propositions for Up-scaling.

[bib13] Carter M.R., Cheng L., Sarris A. (2016). Where and how index insurance can boost the adoption of improved agricultural technologies. J. Dev. Econ..

[bib14] Clarke D.J. (2016). A theory of rational demand for index insurance. Am. Econ. J. Microecon..

[bib15] Clarke D.J. (2011). Insurance Design for Developing Countries.

[bib16] Clarke D., de Nicola F., Hill R.V., Kumar N., Mehta P. (2015). A chat about insurance: experimental results from rural Bangladesh. Appl. Econ. Perspect. Pol..

[bib17] Cole S., Giné X., Tobacman J., Topalova P., Townsend R., Vickery J. (2013). Barriers to household risk management: evidence from India. Am. Econ. J. Appl. Econ..

[bib18] Cole S., Giné X., Vickery J. (2013). How Does Risk Management Influence Production Decisions? Evidence from a Field Experiment.

[bib19] Cole S., Stein D., Tobacman J. (2014). Dynamics of demand for index insurance: evidence from a long-run field experiment. Am. Econ. Rev..

[bib20] de Nicola F. (2015). The impact of weather insurance on consumption, investment, and welfare. Quant. Econ..

[bib21] Delavallade C., Dizon F., Hill R.V., Petraud J.P. (2015). Managing Risk with Insurance and Savings: Experimental Evidence for Male and Female Farm Managers in West Africa.

[bib22] Duflo E., Kremer M., Robinson J. (2011). Nudging farmers to use fertilizer: theory and experimental evidence from Kenya. Am. Econ. Rev..

[bib23] Elabed G., Carter M.R. (2015). Ex-ante impacts of agricultural insurance: Evidence from a field experiment in Mali.

[bib24] Epley N., Mak D., Idson L.C. (2006). Bonus or rebate?: the impact of income framing on spending and saving. J. Behav. Decis. Making.

[bib25] Food and Agricultural Organization of the United Nations (2018). FAOSTAT Database. http://www.fao.org/faostat/en/data/PM.

[bib26] Frison L., Pocock S.L. (1992). Repeated measures in clinical trials: analysis using mean summary statistics and its implications for design. Stat. Med..

[bib27] Giné X., Yang D. (2009). Insurance, credit, and technology adoption: field experimental evidence from Malawi. J. Dev. Econ..

[bib28] Giné X., Townsend R., Vickery J. (2008). Patterns of rainfall insurance participation in rural India. World Bank Econ. Rev..

[bib29] Gunnsteinsson S. (2017). Experimental Identification of Asymmetric Information: Evidence on Crop Insurance in the Philippines.

[bib30] Hazell P.B. (1992). The appropriate role of agricultural insurance in developing countries. J. Int. Dev..

[bib31] Hazell P., Pomareda C., Valdés A. (1986). Crop Insurance for Agricultural Development.

[bib32] Hazell P., Anderson J., Balzer N., Hastrup Clemensen A., Hess U., Rispoli F. (2010). The Potential for Scale and Sustainability in Weather Index Insurance for Agriculture and Rural Livelihoods.

[bib33] Hill R.V., Hoddinott J., Kumar N. (2013). Adoption of weather-index insurance: learning from willingness to pay among a panel of households in rural Ethiopia. Agric. Econ..

[bib34] Hill R.V., Robles M., Ceballos F. (2016). Demand for a simple weather insurance product in India: theory and evidence. Am. J. Agric. Econ..

[bib35] Imbens G.W., Angrist J.D. (1994). Identification and estimation of local average treatment effects. Econometrica.

[bib36] Ito S., Kono H. (2010). Why is take-up of microinsurance so low? Evidence from a health insurance scheme in India. Develop. Econ..

[bib37] J-PAL, CEGA, ATAI (2016). Make it Rain. Policy Bulletin, Abdul Latif Jameel Poverty Action Lab.

[bib38] Janzen S.A., Carter M.R. (2013). After the Drought: the Impact of Microinsurance on Consumption Smoothing and Asset Protection.

[bib39] Just R.E., Calvin L., Quiggin J. (1999). Adverse selection in crop insurance: actuarial and asymmetric information incentives. Am. J. Agric. Econ..

[bib40] Karlan D., Morduch J., Rodrik D., Rosenzweig M. (2009). Access to finance.

[bib41] Karlan D., Osei R., Osei-Akoto I., Udry C. (2014). Agricultural decisions after relaxing credit and risk constraints. Q. J. Econ..

[bib42] McKenzie D. (2012). Beyond baseline and follow-up: the case for more T in experiments. J. Econ. Dev..

[bib43] Miranda M.J. (1991). Area-yield crop insurance reconsidered. Am. J. Agric. Econ..

[bib44] Mobarak A.M., Rosenzweig M.R. (2012). Selling Formal Insurance to the Informally Insured.

[bib45] Mobarak A.M., Rosenzweig M.R. (2013). Informal risk sharing, index insurance, and risk taking in developing countries. Am. Econ. Rev..

[bib46] Morduch J., Bannerjee A., Benabou R., Mookherjee D. (2006). Microinsurance: the next revolution?. Understanding Poverty.

[bib47] Platteau J.-P., De Bock O., Gelade W. (2017). The demand for microinsurance: a literature review. World Dev..

[bib48] Quiggin J. (1992). Some observations on insurance, bankruptcy and input demand. J. Econ. Behav. Organ..

[bib49] Ramamasy S., Baas S. (2007). Climate Variability and Change: Adaptation to Drought in Bangladesh.

[bib50] Ramaswami B. (1992). Production risk and optimal input decisions. Am. J. Agric. Econ..

[bib51] Rosenzweig M.R., Binswanger H.P. (1993). Wealth, weather risk and the composition and profitability of agricultural investments. Econ. J..

[bib52] Sandmo A. (1971). The competitive firm under output price uncertainty. Am. Econ. Rev..

[bib53] Serfilippi E., Carter M., Guirkinger C. (2016). Certain and Uncertain Utility and Insurance Demand: Results from a Framed Field Experiment in Burkina Faso.

[bib55] Van Breukelen G.J. (2006). ANCOVA versus change from baseline had more power in randomized studies and more bias in nonrandomized studies. J. Clin. Epidemiol..

[bib56] Ward P.S., Makhija S. (2018). New modalities for managing drought risk in rainfed agriculture: evidence from a discrete choice experiment in Odisha, India. World Dev..

