# Supplementary material for: Ex ante and ex post effects of hybrid index insurance in Bangladesh
Source: J Dev Econ. 2019 Jan;136:1–17. doi: 10.1016/j.jdeveco.2018.09.003 (PMC6472668; doi:10.1016/j.jdeveco.2018.09.003)
Supplement: Multimedia component 2 [file mmc2.pdf]

A Supplemental tables and figures

Table A1: Timeline of research and agricultural activities during study period

	2013								2014						
**Activity**	**May**	**June**	**July**	**Aug**	**Sept**	**Oct**	**Nov**	**Dec**	**Jan**	**Feb**	**Mar**	**Apr**	**May**	**June**	**July**
Baseline survey															
Insurance marketing															
Insurance coverage period															
Insurance payouts delivered															
Aman season															
Nursery preparation															
Land preparation															
Transplanting															
Flowering															
Harvest															
Boro season															
Nursery preparation															
Land preparation															
Transplanting															
Flowering															
Harvest															
Follow-up survey															

Source: Authors.
Note: Agricultural timeline based on focus group discussions carried out prior to the initiation of the study and reflecting consensus opinions of focus group members. Due to various factors, including weather conditions, soil variability, seed varieties, labor supply constraints, and so on, the timing of agricultural activities and crop growth cycles varies.

Table A2: Insurance policy strike points

Event	Triggers	Description of trigger	Payout
First	14 day dry spell	Maximum number of consecutive dry days when the rainfall recorded at the station is less than or equal to 2 mm in the coverage period is 14 or more days	BDT 600
Second	12 day dry spell	Maximum number of consecutive dry days when the rainfall recorded at the station is less than or equal to 2 mm in the coverage period is 12 or 13 days	BDT 300
Third	Average yield in the *upazila* is less than or equal to 26 maunds per acre	Average yield (as estimated by crop cutting experiment conducted at *upazila* by the Bangladesh Bureau of Statistics) is less than or equal to 26 maunds per acre	BDT 300

Source: Authors.

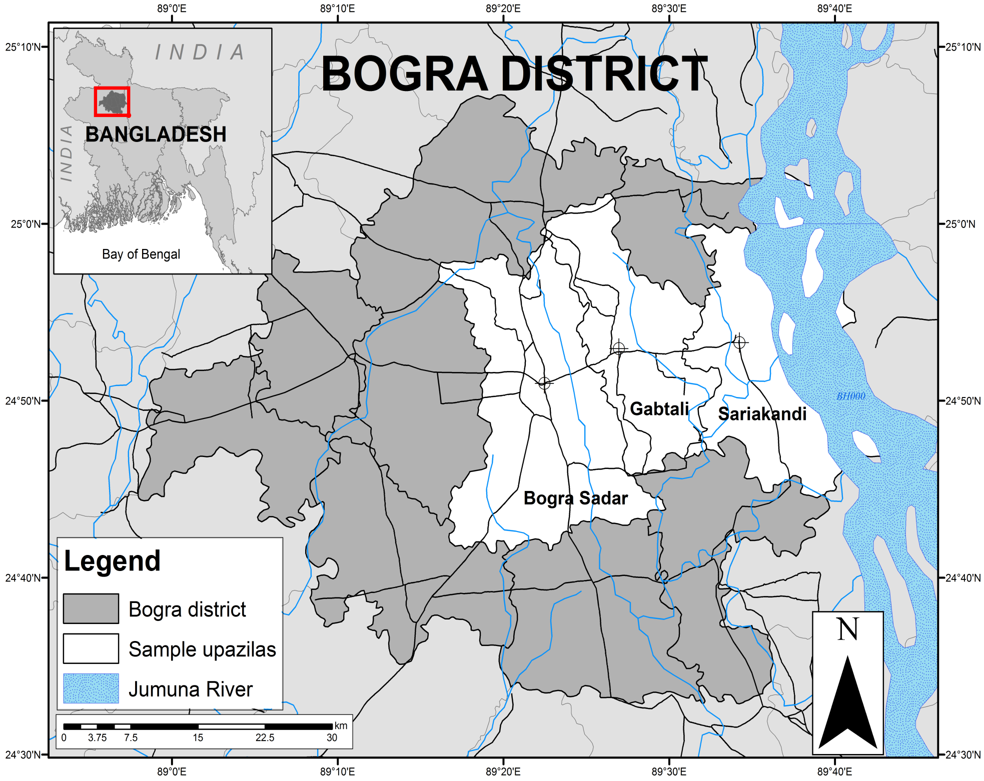

Figure A1: Location of sample *upazilas* in Bogra district, Rajshahi division, Bangladesh

Source: Authors.

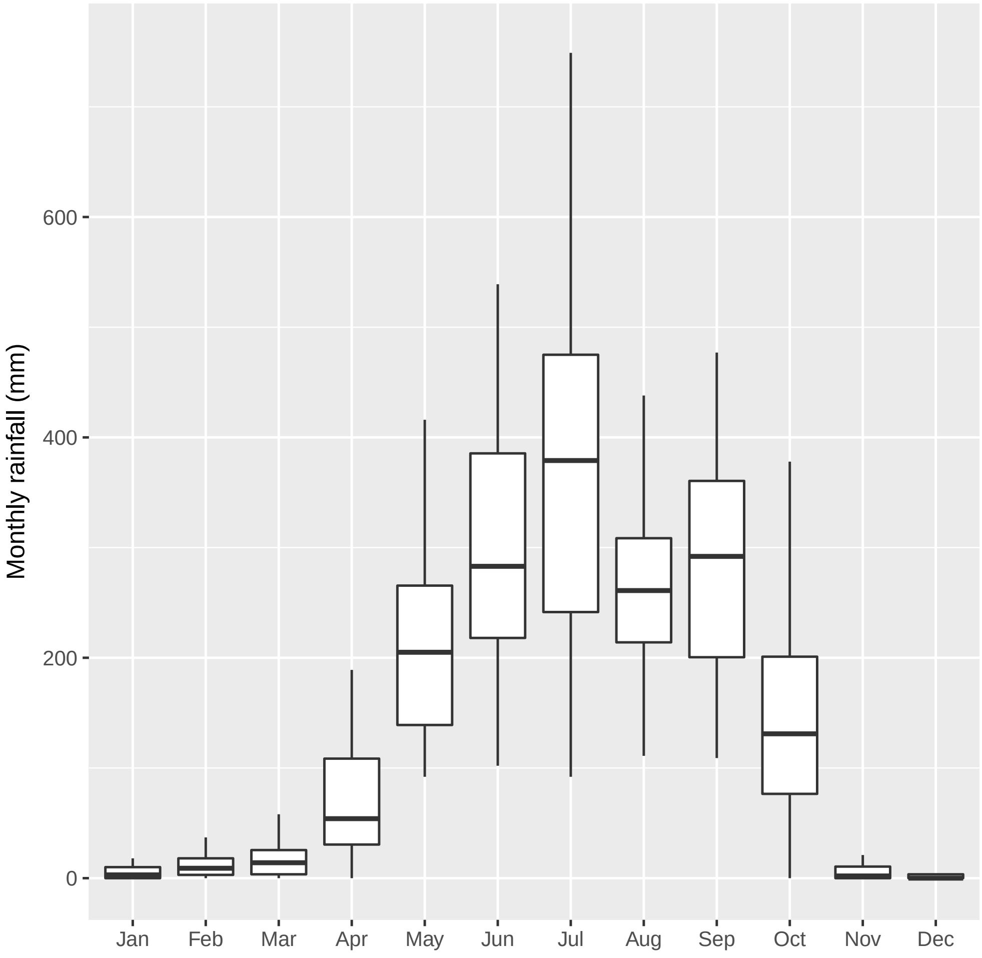

Figure A2: Historical distribution of rainfall by month, Bogra district, Bangladesh

Source: Authors; based on rainfall data from the Bangladesh Meteorological Department weather station in Bogra district, 1980–2010.
Note:

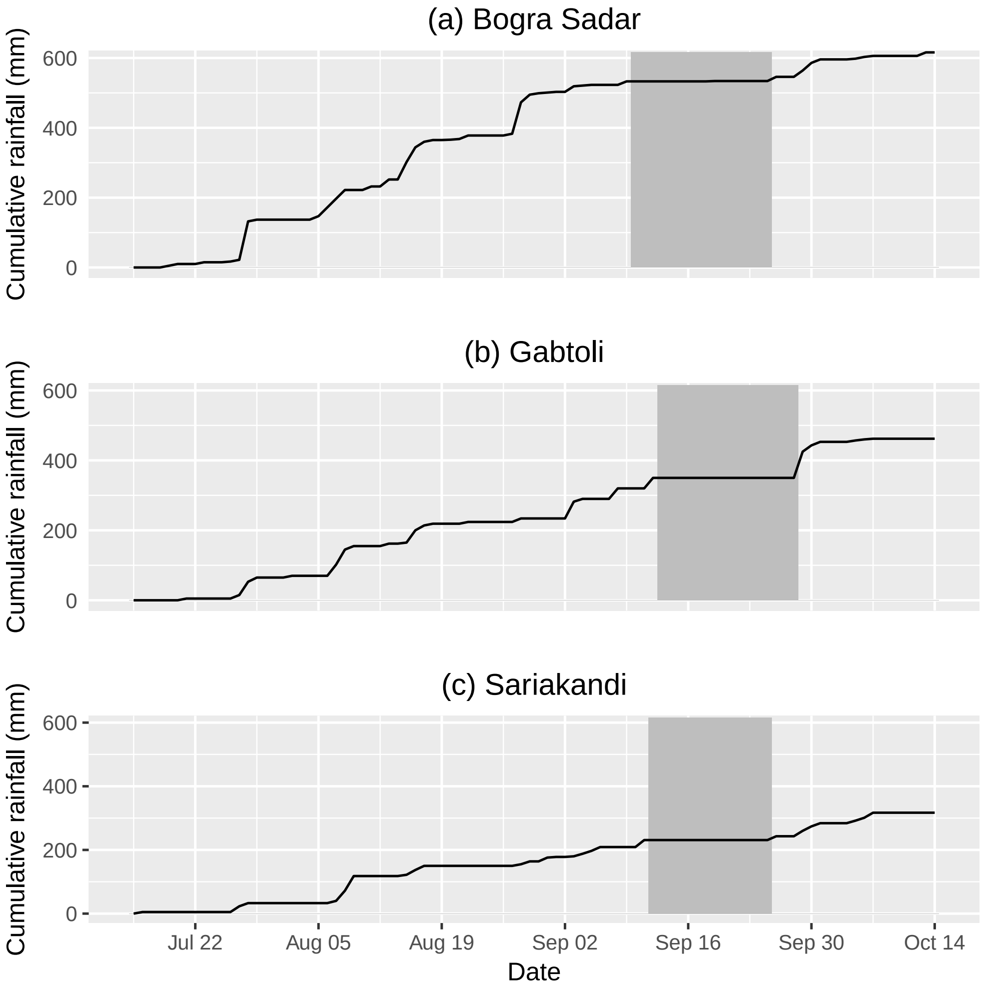

Figure A3: Cumulative rainfall during insurance coverage period (July 15 – October 14, 2013), by *upazila*

Source: Authors; based on data from *upazila* agricultural extension office for Bogra Sadar (top panel), Gabtoli (middle panel), and Sariakandi (bottom panel) *upazilas*.
Note:Grey bars indicate the maximum dry spell recorded in each *upazila*.

B First-stage regressions for primary LATE and dose response regression tables

Table B1: First-stage regressions for LATE regressions in Table 4. Dependent variable: purchased insurance (=1)

	Dependent variable in second stage:									
	Agricultural input expenditures during the dry season (BDT)									
	Irrigation	Pesticides	Fertilizer	Hired labor	Purchased seeds	Total	Total area cultivated (decimals)	Area cultivated with rice (decimals)	Quantity of rice harvested (kg)	Rice yield (kg/decimal)
Intercept	0.017	0.018	0.023	0.024	0.018	0.023	0.017	0.018	0.016	0.025
	(0.040)	(0.039)	(0.039)	(0.040)	(0.040)	(0.040)	(0.040)	(0.040)	(0.040)	(0.040)
Baseline level of second stage dependent variable	0.001	−0.075***	−0.015***	−0.009	−0.010	−0.005***	0.000	−0.000	−0.000	−0.001
	(0.007)	(0.024)	(0.005)	(0.005)	(0.014)	(0.002)	(0.000)	(0.000)	(0.000)	(0.001)
Gender of household head (male = 1)	-0.024	−0.023	−0.022	−0.028	−0.023	−0.025	−0.024	−0.024	−0.023	−0.023
	(0.020)	(0.020)	(0.020)	(0.020)	(0.020)	(0.020)	(0.020)	(0.020)	(0.020)	(0.020)
Household size	0.000	0.001	0.001	0.001	0.000	0.001	0.000	0.000	0.000	0.000
	(0.004)	(0.004)	(0.004)	(0.004)	(0.004)	(0.004)	(0.004)	(0.004)	(0.004)	(0.004)
Total area cultivated during monsoon season (decimals)	0.000	0.000**	0.000**	0.000	0.000	0.000**		0.000	0.000	0.000
	(0.000)	(0.000)	(0.000)	(0.000)	(0.000)	(0.000)		(0.000)	(0.000)	(0.000)
Years household has been affiliated with GUK	0.001	0.000	0.000	0.001	0.001	0.001	0.001	0.000	0.000	0.000
	(0.002)	(0.002)	(0.002)	(0.002)	(0.002)	(0.002)	(0.002)	(0.002)	(0.002)	(0.002)
Cash savings is adequate (yes = 1)	-0.028 **	−0.028**	−0.028**	−0.026**	−0.028**	−0.026**	−0.028**	−0.028**	−0.026**	−0.026**
	(0.013)	(0.013)	(0.013)	(0.013)	(0.013)	(0.013)	(0.013)	(0.013)	(0.013)	(0.013)
Household head is ambiguity-averse (yes = 1)	-0.021 **	−0.018**	−0.019**	−0.019**	−0.021**	−0.018**	−0.021**	−0.020**	−0.020**	−0.020**
	(0.009)	(0.009)	(0.009)	(0.009)	(0.009)	(0.009)	(0.009)	(0.009)	(0.009)	(0.009)
Trust in GUK management	0.007	0.007	0.006	0.006	0.008	0.006	0.007	0.008	0.008	0.008
	(0.009)	(0.009)	(0.009)	(0.009)	(0.009)	(0.009)	(0.009)	(0.009)	(0.009)	(0.009)
Treatment allocation (=1)	0.873 ***	0.873***	0.873***	0.873***	0.873***	0.873***	0.873***	0.873***	0.874***	0.874***
	(0.021)	(0.020)	(0.021)	(0.021)	(0.021)	(0.021)	(0.021)	(0.021)	(0.021)	(0.021)
Number of observations	1977	1977	1977	1977	1977	1977	1977	1977	1977	1977
Adjusted R^2	0.766	0.769	0.768	0.767	0.767	0.768	0.767	0.767	0.767	0.767

Source: Authors.

Note: * Significant at 10 percent level; ** Significant at 5 percent level; *** Significant at 1 percent level. Standard errors adjusted for clustering at the village level in parentheses. Excluded instrument is random assignment into the treatment group.

Table B2: First-stage regressions for dose response regressions in Table 4. Dependent variable: Number of insurance units purchased

	Dependent variable in second stage:									
	Agricultural input expenditures during the dry season (BDT)									
	Irrigation	Pesticides	Fertilizer	Hired labor	Purchased seeds	Total	Total area cultivated (decimals)	Area cultivated with rice (decimals)	Quantity of rice harvested (kg)	Rice yield (kg/decimal)
Intercept	-0.631	−0.635	−0.636	−0.641	−0.650	−0.650	−0.635	−0.625	−0.659	−0.430
	(0.574)	(0.568)	(0.577)	(0.593)	(0.566)	(0.574)	(0.568)	(0.570)	(0.570)	(0.626)
Baseline level of second stage dependent variable	0.018	0.294	0.002	0.008	0.331**	0.015	0.002*	−0.003	−0.000*	−0.024**
	(0.092)	(0.267)	(0.062)	(0.062)	(0.164)	(0.022)	(0.001)	(0.003)	(0.000)	(0.012)
Gender of household head (male = 1)	-0.078	−0.081	−0.078	−0.074	−0.098	−0.074	−0.078	−0.077	−0.065	−0.040
	(0.327)	(0.327)	(0.326)	(0.332)	(0.331)	(0.328)	(0.327)	(0.328)	(0.329)	(0.322)
Household size	0.094 *	0.093*	0.094*	0.094*	0.095*	0.094*	0.094*	0.092*	0.093*	0.095*
	(0.053)	(0.052)	(0.052)	(0.051)	(0.052)	(0.052)	(0.052)	(0.052)	(0.052)	(0.052)
Total area cultivated during monsoon season (decimals)	0.002	0.001	0.002	0.002	0.001	0.001		0.004*	0.005**	0.002*
	(0.001)	(0.001)	(0.002)	(0.002)	(0.001)	(0.002)		(0.002)	(0.002)	(0.001)
Years household has been affiliated with GUK	0.017	0.018	0.017	0.017	0.014	0.017	0.017	0.016	0.015	0.015
	(0.028)	(0.029)	(0.028)	(0.028)	(0.028)	(0.028)	(0.028)	(0.028)	(0.028)	(0.028)
Cash savings is adequate (yes = 1)	-0.219	−0.218	−0.218	−0.220	−0.235	−0.223	−0.218	−0.216	−0.186	−0.169
	(0.180)	(0.180)	(0.180)	(0.175)	(0.182)	(0.179)	(0.180)	(0.179)	(0.167)	(0.162)
Household head is ambiguity-averse (yes = 1)	-0.020	−0.029	−0.019	−0.020	−0.016	−0.027	−0.019	−0.008	0.003	−0.004
	(0.112)	(0.106)	(0.108)	(0.107)	(0.112)	(0.106)	(0.112)	(0.117)	(0.112)	(0.110)
Trust in GUK management	0.036	0.039	0.038	0.039	0.033	0.043	0.037	0.038	0.042	0.051
	(0.115)	(0.114)	(0.115)	(0.115)	(0.112)	(0.113)	(0.113)	(0.112)	(0.111)	
Incentive level	0.037 ***	0.037***	0.037***	0.037***	0.037***	0.037***	0.037***	0.037***	0.038***	0.038***
	(0.007)	(0.007)	(0.007)	(0.007)	(0.007)	(0.007)	(0.007)	(0.007)	(0.007)	
Number of observations	1977	1977	1977	1977	1977	1977	1977	1977	1977	1977
Adjusted R^2	0.176	0.178	0.176	0.176	0.179	0.177	0.177	0.176	0.178	0.178

Source: Authors.

Note: * Significant at 10 percent level; ** Significant at 5 percent level; *** Significant at 1 percent level. Standard errors adjusted for clustering at the village level in parentheses. Excluded instrument is the level of the incentive.

Table B3: First-stage regressions for LATE regressions in Table 5. Dependent variable: purchased insurance (=1)

	Dependent variable in second stage:									
	Agricultural input expenditures during the dry season (BDT)									
	Irrigation	Pesticides	Fertilizer	Hired labor	Purchased seeds	Total	Total area cultivated (decimals)	Area cultivated with rice (decimals)	Quantity of rice harvested (kg)	Rice yield (kg/decimal)
Intercept	0.022	0.024	0.021	0.023	0.024	0.023	0.024	0.021	0.025	0.033
	(0.040)	(0.040)	(0.040)	(0.040)	(0.040)	(0.040)	(0.040)	(0.039)	(0.040)	(0.038)
Baseline level of second stage dependent variable	0.004	−0.044***	−0.004	−0.006	−0.007	−0.002	−0.000	0.000*	0.000	−0.001
	(0.003)	(0.013)	(0.003)	(0.004)	(0.007)	(0.001)	(0.000)	(0.000)	(0.000)	(0.001)
Gender of household head (male = 1)	-0.022	−0.023	−0.021	−0.026	−0.023	−0.023	−0.023	−0.023	−0.024	−0.022
	(0.020)	(0.020)	(0.020)	(0.020)	(0.020)	(0.020)	(0.020)	(0.020)	(0.020)	(0.020)
Household size	0.002	0.001	0.002	0.002	0.002	0.002	0.002	0.002	0.002	0.002
	(0.004)	(0.004)	(0.004)	(0.004)	(0.004)	(0.004)	(0.004)	(0.004)	(0.004)	(0.004)
Total area cultivated during dry season (decimals)	-0.000 *	0.000	0.000	0.000	−0.000	0.000		−0.000**	−0.000*	−0.000
	(0.000)	(0.000)	(0.000)	(0.000)	(0.000)	(0.000)		(0.000)	(0.000)	(0.000)
Years household has been affiliated with GUK	0.000	0.000	0.000	0.001	0.001	0.001	0.001	0.001	0.001	0.001
	(0.002)	(0.002)	(0.002)	(0.002)	(0.002)	(0.002)	(0.002)	(0.002)	(0.002)	(0.002)
Cash savings is adequate (yes = 1)	-0.025 *	−0.021*	−0.024*	−0.023*	−0.024*	−0.024*	−0.026*	−0.025*	−0.026**	−0.025*
	(0.013)	(0.013)	(0.013)	(0.013)	(0.013)	(0.013)	(0.013)	(0.013)	(0.013)	(0.013)
Household head is ambiguity-averse (yes = 1)	-0.021 **	−0.018**	−0.019**	−0.019**	−0.020**	−0.019**	−0.020**	−0.021**	−0.021**	−0.020**
	(0.009)	(0.009)	(0.009)	(0.009)	(0.009)	(0.009)	(0.009)	(0.009)	(0.009)	(0.009)
Trust in GUK management	0.006	0.007	0.007	0.007	0.007	0.007	0.007	0.006	0.006	0.007
	(0.009)	(0.009)	(0.009)	(0.009)	(0.009)	(0.009)	(0.009)	(0.009)	(0.009)	(0.009)
Treatment allocation (=1)	0.872 ***	0.872***	0.873***	0.872***	0.873***	0.873***	0.873***	0.873***	0.872***	0.873***
	(0.021)	(0.021)	(0.021)	(0.021)	(0.021)	(0.021)	(0.021)	(0.021)	(0.021)	(0.021)
Number of observations	1977	1977	1977	1977	1977	1977	1977	1977	1977	1977
Adjusted R^2	0.767	0.769	0.767	0.768	0.767	0.767	0.767	0.767	0.767	0.767

Source: Authors.

Note: * Significant at 10 percent level; ** Significant at 5 percent level; *** Significant at 1 percent level. Standard errors adjusted for clustering at the village level in parentheses. Excluded instrument is random assignment into the treatment group.

Table B4: First-stage regressions for dose response regressions in Table 5. Dependent variable: Number of insurance units purchased

	Dependent variable in second stage:									
	Agricultural input expenditures during the dry season (BDT)									
	Irrigation	Pesticides	Fertilizer	Hired labor	Purchased seeds	Total	Total area cultivated (decimals)	Area cultivated with rice (decimals)	Quantity of rice harvested (kg)	Rice yield (kg/decimal)
Intercept	-0.685	−0.633	−0.621	−0.637	−0.638	−0.641	−0.653	−0.686	−0.611	−0.640
	(0.565)	(0.570)	(0.564)	(0.569)	(0.571)	(0.566)	(0.575)	(0.568)	(0.564)	(0.522)
Baseline level of second stage dependent variable	0.149 **	−0.041	0.039*	0.013	0.044	0.020*	0.002	0.007**	0.000**	0.000
	(0.059)	(0.091)	(0.021)	(0.023)	(0.061)	(0.010)	(0.001)	(0.004)	(0.000)	(0.010)
Gender of household head (male = 1)	-0.042	−0.078	−0.092	−0.069	−0.076	−0.071	−0.078	−0.074	−0.095	−0.078
	(0.319)	(0.328)	(0.327)	(0.322)	(0.330)	(0.327)	(0.329)	(0.323)	(0.323)	(0.325)
Household size	0.074	0.095*	0.087*	0.092*	0.093*	0.082*	0.087*	0.079	0.079	0.094*
	(0.051)	(0.052)	(0.050)	(0.050)	(0.052)	(0.050)	(0.049)	(0.049)	(0.051)	(0.051)
Total area cultivated during dry season (decimals)	-0.002	0.002**	0.000	0.002	0.002	−0.001		−0.003	−0.003	0.002*
	(0.002)	(0.001)	(0.001)	(0.001)	(0.001)	(0.002)		(0.003)	(0.003)	(0.001)
Years household has been affiliated with GUK	0.015	0.017	0.017	0.017	0.016	0.016	0.017	0.019	0.019	0.017
	(0.028)	(0.028)	(0.028)	(0.028)	(0.027)	(0.028)	(0.028)	(0.028)	(0.028)	(0.028)
Cash savings is adequate (yes = 1)	-0.204	−0.213	−0.238	−0.224	−0.231	−0.246	−0.226	−0.215	−0.242	−0.218
	(0.174)	(0.180)	(0.179)	(0.180)	(0.176)	(0.185)	(0.181)	(0.178)	(0.184)	(0.184)
Household head is ambiguity-averse (yes = 1)	-0.025	−0.016	−0.033	−0.022	−0.021	−0.040	−0.021	−0.039	−0.043	−0.019
	(0.108)	(0.111)	(0.111)	(0.111)	(0.111)	(0.111)	(0.110)	(0.108)	(0.109)	(0.116)
Trust in GUK management	0.026	0.037	0.044	0.039	0.035	0.045	0.047	0.052	0.039	0.037
	(0.112)	(0.114)	(0.113)	(0.115)	(0.114)	(0.117)	(0.115)	(0.114)	(0.118)	
Incentive level	0.037 ***	0.037***	0.037***	0.037***	0.037***	0.037***	0.037***	0.037***	0.037***	0.037***
	(0.007)	(0.007)	(0.007)	(0.007)	(0.007)	(0.007)	(0.007)	(0.007)	(0.007)	
Number of observations	1977	1977	1977	1977	1977	1977	1977	1977	1977	1977
Adjusted R^2	0.188	0.178	0.178	0.178	0.178	0.180	0.178	0.178	0.179	0.180

Source: Authors.

Note: * Significant at 10 percent level; ** Significant at 5 percent level; *** Significant at 1 percent level. Standard errors adjusted for clustering at the village level in parentheses. Excluded instrument is the level of the incentive.

C Impact regressions with treatment restricted to (A) only the subgroup receiving discounts or (B) only the subgroup receiving rebates

Table C1: Intention-to-treat effects, local average treatment effects, and dose responses of index insurance on agricultural input use and *aman* rice production (monsoon season)

			Agricultural input expenditures during the monsoon season (BDT)									
			Irrigation	Pesticides	Fertilizer	Hired labor	Purchased seeds	Total	Total area cultivated (decimals)	Area cultivated with rice (decimals)	Quantity of rice harvested (kg)	Rice yield (kg/decimal)
(A)	(1)	Intention to treat effect (ITT)	387.810***	76.868**	637.417***	376.454*	44.787	1838.271***	12.239***	−1.373	−73.970	−1.415
			(99.069)	(34.091)	(195.461)	(216.618)	(59.718)	(595.434)	(3.516)	(3.543)	(63.252)	(0.896)
		Adjusted R^2	0.280	0.253	0.337	0.336	0.062	0.400	0.525	0.389	0.368	0.039
	(2)	Local average treatment effect (LATE)	429.338***	85.093**	705.875***	416.785*	49.583	2034.193***	13.550***	−1.520	−81.884	−1.566
			(109.464)	(38.165)	(221.207)	(243.108)	(66.355)	(673.196)	(3.989)	(3.926)	(70.193)	(0.996)
		Adjusted R^2	0.275	0.249	0.324	0.333	0.060	0.395	0.519	0.370	0.365	0.035
	(3)	Dose response effect	86.619**	21.259**	180.317***	95.351	18.000	481.013***	3.187***	−0.226	−15.884	−0.322
			(34.240)	(9.438)	(58.211)	(58.795)	(14.960)	(181.507)	(1.141)	(0.876)	(15.896)	(0.239)
		Adjusted R^2	0.228	0.233	0.296	0.322	0.056	0.369	0.382	0.369	0.361	0.021
		Observations	1478	1478	1478	1478	1478	1478	1478	1478	1478	1478
		Mean for comparison group at endline	866.404	295.638	2270.487	2217.143	364.788	7516.416	65.728	44.297	756.047	13.622
		Mean for treatment group at endline	1309.732	389.006	2971.513	2680.022	435.335	9698.022	79.877	43.872	716.449	12.316
		Unadjusted ITT effect	448.781***	94.747**	702.938***	470.460	74.542	2209.509	14.302**	−0.464	−40.578	−1.314
		(133.637) (46.242)	(257.104)	(289.103)	(64.196)	(819.522)	(5.721)	(4.758)	(83.869)	(0.933)		
(B)	(1)	Intention to treat effect (ITT)	205.875**	71.734*	452.911**	568.805**	100.339	1621.182**	9.206**	3.931	27.873	0.189
			(102.868)	(38.389)	(192.018)	(238.024)	(71.373)	(685.312)	(4.232)	(2.881)	(58.400)	(0.740)
		Adjusted R^2	0.231	0.250	0.336	0.335	0.066	0.382	0.501	0.403	0.385	0.060
	(2)	Local average treatment effect (LATE)	244.854**	85.289*	538.700**	676.175**	119.243	1927.418**	10.948**	4.673	32.969	0.225
			(122.065)	(46.827)	(233.417)	(291.227)	(87.342)	(836.660)	(5.194)	(3.428)	(69.067)	(0.880)
		Adjusted R^2	0.231	0.242	0.330	0.333	0.060	0.377	0.496	0.403	0.385	0.060
	(3)	Dose response effect	119.630	64.304*	397.272**	480.089**	65.884	1337.748**	7.935**	3.908	36.594	0.394
			(81.810)	(33.526)	(162.916)	(204.130)	(60.122)	(592.995)	(3.454)	(2.017)	(42.696)	(0.617)
		Adjusted R^2	0.229	0.242	0.335	0.329	0.060	0.379	−13.310	0.403	0.385	0.059
		Observations	1476	1476	1476	1476	1476	1476	1476	1476	1476	1476
		Mean for comparison group at endline	866.404	295.638	2270.487	2217.143	364.788	7516.416	65.728	44.297	756.047	13.622
		Mean for treatment group at endline	1050.300	352.655	2606.230	2643.287	462.529	8766.537	71.169	45.813	767.024	13.975
		Unadjusted ITT effect	187.106	58.841	339.052	428.995	100.031	1266.088	5.480	1.508	10.612	0.350
			(138.427)	(53.543)	(262.817)	(327.212)	(78.714)	(925.206)	(6.056)	(4.194)	(83.426)	(0.854)

Source: Authors.

Note: * Significant at 10 percent level; ** Significant at 5 percent level; *** Significant at 1 percent level. Panel (A) reports regressions with treatment group restricted only to those households receiving a discount on the cost of insurance. Panel (B) reports regressions with treatment group restricted only to those households receving a rebate. In LATE regression, binary variable indicating random assignment to the treatment group serves as an instrument for insurance take-up. In dose response regression, the level of the incentive serves as an instrument for the insurance coverage amount. Standard errors adjusted for clustering at the village level in parentheses. For LATE and dose response regressions, standard errors have been adjusted for clustering at the village level in both the first and second stages. ITT, LATE, and dose response regressions control for the baseline level of the outcome variable as well as farm size (total area cultivated during during monsoon season at baseline) and household and agricultural characteristics for which there was an imbalance between treatment and comparison groups at baseline. Unadjusted ITT regressions report mean differences in levels of outcomes between treatment and comparison at endline without controlling for baseline levels of outcome variables or characteristics for which there were imbalances at baseline.
